# Dynamic, single-cell monitoring of CAR T cell identity and activation with Raman spectroscopy

**DOI:** 10.64898/2026.02.22.707331

**Published:** 2026-02-23

**Authors:** Ariel Stiber, Boi Quach, Babatunde Ogunlade, Antony Georgiadis, Kai Chang, Yuanwei Li, Patrick Quinn, Haoqing Wang, Kristin C. Y. Tsui, Charm Ang, Elena Sotillo, Crystal Mackall, Zinaida Good, Jennifer A. Dionne

**Affiliations:** 1 Department of Materials Science and Engineering, Stanford University, Stanford, California, USA; 2 Division of Immunology and Rheumatology, Department of Medicine, Stanford University School of Medicine, Stanford, California, USA; 3 Division of Computational Medicine, Department of Medicine, Stanford University School of Medicine, Stanford, California, USA; 4 Center for Cancer Cell Therapy, Stanford Cancer Institute, Stanford University, Stanford, California, USA; 5 Department of Electrical Engineering, Stanford University, Stanford, California, USA; 6 Stanford Cryo-EM Microscopy Center, Stanford University, Stanford, California, USA; 7 Cryoelectron Microscopy, Nucleus at Sarafan ChEM-H, Stanford University, Stanford, California, USA; 8 Division of Hematology and Oncology, Department of Pediatrics, Stanford University, Stanford, CA, USA; 9 Division of Blood and Marrow Transplantation and Cellular Therapy, Department of Medicine, Stanford University, Stanford, California, USA; 10 Parker Institute for Cancer Immunotherapy, Stanford University, Stanford, California, USA; 11 Weill Cancer Hub West, Stanford University, Stanford, California, USA; 12 Department of Radiology, Stanford University School of Medicine, Stanford, California, USA

## Abstract

Chimeric antigen receptor (CAR) T cell therapies have reshaped treatment for cancers and immune-mediated diseases, yet their safety and efficacy depend on both the proliferation of engineered cells and their dynamic functional state — features that remain challenging to monitor in real-time clinical settings. Current methods require labels, extensive processing, and provide only static snapshots of cell identity and activation. Here, we introduce a surface-enhanced Raman spectroscopy and machine learning approach that enables label-free single-cell identification of engineered CAR T cells and time-resolved, semi-continuous monitoring of their functional activation state. Using the intrinsic vibrational signatures from live cells, we detect spectral differences resulting from engineered receptor expression in donor-derived CD19- and GD2-targeted CAR T cells (nine and five donors, respectively) with 81–85% donor-level accuracy and resolve dynamic antigen-specific activation trajectories with temporal precision. These capabilities stem from biochemical signatures consistent with processes such as receptor expression, tonic signalling, and immune synapse formation, demonstrating a single method that reports both cellular identity and activation state with biochemical specificity. Our results extend CAR T cell monitoring beyond static phenotyping and establish the potential of SERS-ML analysis for rapid, point-of-care assessment of engineered immune cells.

## Introduction

Chimeric antigen receptor (CAR) T cell therapy has emerged as a transformative immunotherapy with the potential to improve the treatment of cancer, autoimmune, and inflammatory diseases. In this approach, autologous T lymphocytes are isolated and genetically engineered to express CARs, synthetic receptors that direct cytotoxicity towards target cells expressing a chosen antigen. CAR T cell therapies minimize the collateral damage observed with conventional cancer therapies, such as chemotherapy, and elicit more robust immune responses than those typically achieved by antibody-based therapies. CD19-targeted therapies (CD19-CAR) have already changed the standard of care for refractory B cell and plasma cell malignancies^1^ and are expanding into earlier lines of treatment.^2^ Beyond hematologic malignancies, several CAR constructs have shown encouraging clinical activity in solid tumors, including GD2-CAR T cells for diffuse midline gliomas.^3^ Given their growing use and versatility, CAR T cell therapies have become a pillar of modern medicine.

Several obstacles still limit effective CAR T development, particularly the risk of adverse events and variable efficacy. Post-infusion CAR T cell expansion and persistence are important correlates of efficacy and toxicity.^4,5^ Excessive expansion can trigger cytokine release syndrome (CRS) and immune effector cell-associated neurotoxicity syndrome (ICANS), which develop rapidly but can be mitigated if identified early. Conversely, insufficient CAR T cell expansion within the first two weeks post-infusion may indicate treatment failure, necessitating early consideration of alternatives. Further, a co-incidence of fever with a lack of CAR T cell expansion may instead indicate infection, requiring treatment distinct from CRS and ICANS mitigation. Tools to quantify and monitor CAR T cells throughout treatment are therefore vital for both research and clinical care.

Current CAR T cell monitoring methods generally trade off depth of phenotypic cellular information and clinical practicality. Transcriptomic profiling provides detailed molecular information on identity and activation state, but is expensive, slow, labor-intensive, and not routinely performed in clinical workflows.^6^ PCR and flow cytometry are used more commonly; however, PCR does not yield phenotypic information and is not a perfect correlate of circulating cell numbers due to the potential for multiple transgene integration events.^7^ Flow cytometry, on the other hand, is susceptible to batch variability and operator bias, requires pre-planned fluorescent panels and labeling, and can be perturbative to cell function.^8,9^ Additionally, it cannot continuously monitor the phenotypic changes associated with dynamic T cell activation. As a result, CAR T cell monitoring is often time-consuming and expensive, precluding prompt point-of-care support and relegating vital data to retrospective analyses.^10^ Newer label-free techniques, such as ghost cytometry and morphology-based sorting, enable high-throughput cellular phenotyping based on optical properties including size, morphology, and refractive index.^9,11^ However, these methods have not yet been shown to differentiate subtle changes resulting from genetic engineering and, because they primarily capture morphology and optical properties rather than molecular composition, offer limited biochemical specificity. Collectively, these monitoring methods generally offer only static or periodic snapshots of cell states and lack the high temporal resolution and dynamic capabilities needed for real-time analytics.

Here, we present a label-free method for live, single-cell, dynamic CAR T cell quantification and state monitoring using surface-enhanced Raman spectroscopy (SERS) and machine learning (ML). Raman spectroscopy measures inelastic light scattering from molecular vibrations; the vibrational energies of biomolecules serve as intrinsic labels, avoiding exogenous labels or perturbative sample preparation.^12^ In SERS, optically resonant nanostructures amplify local electromagnetic fields under laser illumination, enhancing Raman signals by at least 4 orders of magnitude (and often higher).^13–16^ This increases spatial sensitivity and reduces acquisition times to seconds or sub-seconds. The resulting optical “fingerprints,” when analyzed by an integrated ML algorithm with the ability to recognize patterns in complex datasets, inform identity and real-time responses to various environmental conditions at single-cell resolution. Raman spectroscopy, often combined with ML, has previously been used to classify immune cells^13,17^ and assess activation^18–22^ or drug response,^23^ but has largely focused on chemically stimulated or systemically activated T cells measured at discrete timepoints and has typically compared only static resting versus activated states.

**Fig. 1:**
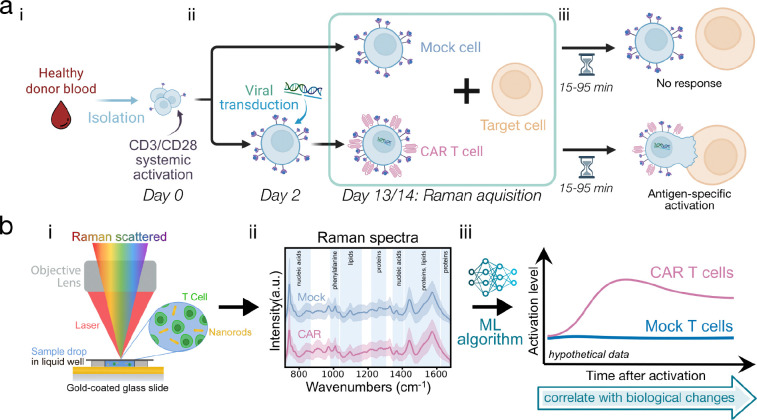
Experimental setup and workflow. **(a)** (i) T cells are isolated from healthy donor blood and activated with CD3/CD28. (ii) They are then separated into “Mock” T cells (further expanded without any introduction to viral vectors) and Chimeric Antigen Receptor (CAR) T cells, which undergo viral transduction to express CAR proteins. (iii) These two cell groups are later co-cultured with target cells expressing the protein that the CAR targets. After an incubation period, an antigen-specific activation response can be observed in the sample with CAR T cells. **(b)** To observe and analyze the cells and processes outlined in (a), we develop a Raman spectroscopy-based platform. (i) Single-cell Raman spectra are collected on a liquid well containing both gold nanorods and live cells. These spectra (ii) are used as input in a machine learning algorithm (iii), where the trained model will distinguish between CAR and Mock cells. All experiments were performed on cells day 13 or 14 after CD3/CD28 activation, and antigen-specific activation Raman measurements were collected 15–95 minutes after initiating co-culture.

We generated donor-derived Mock (untransduced) and CAR T cells (Fig. 1ai–ii) and analyzed them individually and in co-culture with target cells to study how CAR expression and antigen-specific activation shape Raman spectra (Fig. 1aiii). Using SERS (Fig. 1bi), we obtained spectra with key biomolecular features (Fig. 1bii)^24^ and, through ML analysis of these multivariate signatures (Fig. 1biii), demonstrated both cellular identification and functional monitoring. In this more challenging setting, as compared with distinguishing T cells from unrelated cell types, our technique classified CD19-CAR T cells from Mock with ~81% average donor-level accuracy across nine donors, and GD2-CAR T cells from Mock with ~85% accuracy across five donors. Antigen-specific activation in co-culture was distinguished with ~95% accuracy. Semi-continuous tracking of activation dynamics produced time-resolved spectra that aligned with known activation biomarker progression and flow cytometry trends, demonstrating that Raman spectra can capture dynamic functional states. These results show that both CAR expression and activation state produce detectable spectral shifts, notably in protein-, aromatic-, nucleic acid-, and mitochondrial-associated bands. Collectively, our method enables label-free, dynamic phenotyping of live, donor-derived CAR T cells, an important step towards rapid, low-cost, point-of-care monitoring of engineered cell therapies.

## Results

### Nanorods enable rapid live-cell SERS

**Fig. 2:**
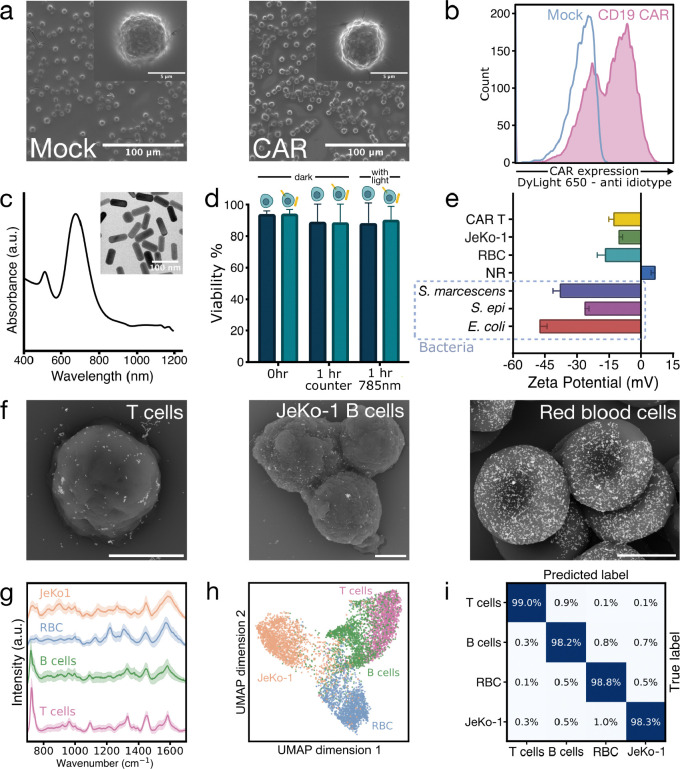
Gold nanorod-enabled surface-enhanced Raman spectroscopy (SERS) of blood cell types. **(a)** Scanning electron micrographs of Au/Pd-coated, dried Mock (left) and CAR (right) T cells. **(b)** Flow cytometry of CD19-CAR expression in healthy human donor T cells compared to Mock. **(c)** Transmission electron micrograph of gold nanorods (left) and their localized surface plasmon resonance peak at ~670 nm (right). **(d)** Cell viability of cells incubated with and without nanorods and exposed to laser illumination for 1 hour. Conditions were performed in duplicate (n=2), with mean and standard deviation. **(e)** Surface charge (zeta potential) of blood cells, bacteria, and gold nanorods. **(f)** Scanning electron micrographs of Au/Pd-coated and dried T, JeKo-1 B, and red blood cells mixed with nanorods (lighter contrast features). Scale bars indicate 3 μm. **(g)** Mean normalized single-cell Raman spectra of live T, primary B, JeKo-1 B, and red blood cells with ±1 standard deviations (SD) (shaded). **(h)** Two-dimensional Uniform Manifold Approximation and Projection (UMAP) of Raman spectra, balanced by class. **(i)** Normalized confusion matrix displaying classification accuracy for these Raman spectra (non-reduced), generated by a Light Gradient-Boosting Machine (LightGBM) classifier with 10-fold stratified cross-validation.

We generated CAR T cells from healthy donor T cells using standard activation and retroviral transduction protocols (Fig. 1a). T cells were transduced with vectors encoding either CD19.28ζ (generating CD19-CAR T cells) or GD2.4–1BBζ (generating GD2-CAR T cells).^3,25^ As a negative control, donor-matched Mock T cells were generated under the same activation and expansion conditions, but without viral transduction. Scanning electron micrographs (SEMs) of fixed, dried CAR and Mock T cells showed no major morphological differences (Fig. 2a). We assessed CAR expression by flow cytometry (Fig. 2b). Transduction efficiencies varied by donor, ranging from 55–96% for CD19-CAR T cells and 35–41% for GD2-CAR T cells (Supplementary Table 1), consistent with reported clinical manufacturing ranges of ~30–70%.^26^

Gold nanorods were selected for live-cell SERS because of their optical tunability, broad biocompatibility, and scalable synthesis. Nanorods were synthesized by seed-mediated colloidal growth to exhibit an approximate resonance peak of 680 nm, blue-shifted to minimize extinction losses in liquid under 785 nm excitation (Fig. 2c; Supplementary Fig. 1).^27^ Acridine orange/propidium iodide assays confirmed biocompatibility after 1-hour incubations with or without nanorods and with or without 785 nm illumination (Fig. 2d). A nanorod concentration of ~385 μg/mL (nominal based on stock; Methods) ensured reproducible SERS enhancement through electrostatic association between positively-charged washed nanorods (ζ ≈ +7 mV) and negatively charged mammalian cells (ζ ≈ −14 mV on average; Fig. 2e). This approach avoided ligand-exchange steps that would introduce Raman background. Dried SEM micrographs confirmed nanorod binding to cell surfaces (Fig. 2f), and cryogenic transmission electron microscopy verified this association under liquid conditions (Supplementary Fig. 2; Supplementary Videos 1–3).

Raman spectra were collected from live cells in a sealed liquid well chamber (Fig. 1bi). Under the same acquisition parameters, SERS spectra exhibited substantially higher intensity than non-enhanced measurements, and nanorod-only controls confirmed that dominant spectral features originated from cells (Supplementary Fig. 3). Local variations in nanorod distribution resulted in expected hotspot heterogeneity, with each spectrum capturing a snapshot of the probed cellular microenvironment. Averaging across spectra produced a representation of the overall biochemical composition.

### Human blood cell classification

As an initial demonstration of label-free live cell identification, we acquired ~11,500 single-cell Raman spectra from four cell types — T cells (untransduced and not systemically activated), primary human B cells, mantle cell lymphoma B cell line (JeKo-1), and red blood cells (RBCs), all suspended in phosphate-buffered saline (PBS) (Fig. 2g; Supplementary Table 2; Methods). Uniform Manifold Approximation and Projection (UMAP) visualization of preprocessed spectra identified clustering by cell type (Fig. 2h; Supplementary Fig. 4).^28^ ML models trained on the full non-reduced spectral dataset achieved 98.6% classification accuracy using 10-fold stratified cross-validation (CV) (Fig. 2i; Supplementary Table 3).

Discriminating peaks correspond with known Raman biochemical features resulting from blood cell identity (Supplementary Fig. 5; Supplementary Table 4),^20,29–35^ including those from DNA/RNA (adenine ring breathing 725–730 cm^−1^, DNA backbone PO_2_^−^ stretch 1,095–1,100 cm^−1^), phenylalanine (1,004 cm^−1^), cytochrome c (750 cm^−1^), hemoproteins (1,128, 1,225, 1,565–70, 1,620–25 cm^−1^), general protein bands (1,140–1,160, 1,620–65 cm^−1^), and lipids (1,330, 1,460 cm^−1^). The adenine-associated peak at ~725 cm^−1^ had the largest contribution to classifying T cells, primary B cells, JeKo-1 B cells, and red blood cells (Supplementary Fig. 5). Its reduced presence in both B cell populations relative to T cells is consistent with a smaller nuclear contribution within the sampling volume, potentially influenced by differences in cell size and nucleus-to-cytoplasm geometry. Mature erythrocytes, lacking nuclei and mitochondria, show minimal nucleic acid and cytochrome c signatures but strong hemoprotein bands. Any residual intensity around 724 cm^−1^ in the RBC spectra likely arises from hypoxanthine, which previous SERS studies found accumulates during blood storage.^32^ Relative to T cell spectra, JeKo-1 B cell spectra exhibit more intense peaks associated with phenylalanine and other general proteins. This may partly reflect their larger size, as intensity can reflect protein concentration. Additionally, transformed cells such as JeKo-1 B cells are in a proliferative state, which may contribute to increased protein production.^30^ Overall, primary B cell spectra tend to fall between T and JeKo-1 cells across multiple discriminative features.

### Distinguishing CD19-CAR from Mock T cells

**Fig. 3:**
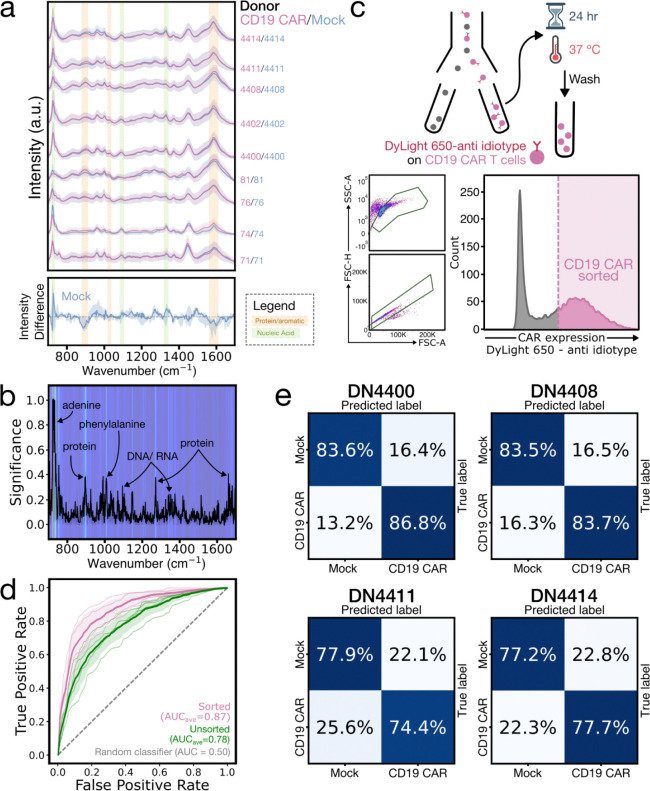
CD19-CAR and Mock T cell SERS classification. **(a)** Waterfall plot of mean normalized SERS spectra of live single CD19-CAR (pink) and Mock (blue) T cells with ±1 SD (shaded), separated by donor (identifier labeled on the right) (top). Median donor-normalized spectral difference of Mock cells relative to CAR with 25–75% quantile range (bottom). The grey dashed line marks zero difference. Colored spectral bands highlight CAR-enriched protein/aromatic (gold) and Mock-enriched nucleic acid (green) regions. Over 2,000 spectra were acquired per donor. **(b)** Mean feature importance plot depicting the contribution of each spectral dimension (wavenumber) to classification, both through the curve and the heatmap background. Significant peaks are labeled with their biochemical vibrational modes. **(c)** Cell sorting schematic (top). CD19-CAR T cells were labeled with DyLight 650-conjugated FMC63 anti-idiotype and flow sorted to achieve a 100% CAR positive sample. After overnight incubation and washing, the labels were effectively removed and cells were analyzed by Raman spectroscopy. Flow cytometry plots show gating for single cells (bottom left) and DyLight 650 labeling intensity (bottom right). **(d)** Receiver operating characteristic (ROC) curves comparing classification performance of unsorted (green) and sorted (pink) CAR T cells versus Mock. Thick lines show the mean across four donors with ±1 SD and thin lines show individual donor curves. The grey dashed line represents a random classifier. Areas under the curve (AUC) are given in the legend. **(e)** Normalized confusion matrices for 4 of 9 donors displaying the classification accuracy for sorted CD19-CAR and Mock Raman spectra using a LightGBM classifier with 10-fold stratified cross-validation.

Having demonstrated blood cell type discrimination, we next asked whether SERS could distinguish more subtle molecular differences arising from T cell engineering. We acquired ~33,000 single-cell spectra from live anti-CD19-CD28-CD3ζ CAR (CD19-CAR; 55–96% transduction efficiency) and donor-matched non-engineered Mock T cells across nine healthy donors (Fig. 3a, top; Methods). Training models on each donor individually achieved an average donor-level 10-fold CV classification accuracy of 75.0% (Supplementary Fig. 6). Analysis of intensity differences (Fig. 3a, bottom) and feature importances (Fig. 3b) revealed that CD19-CAR T cell spectra exhibited consistent enrichment in protein and aromatic bands (880–900, 1,020–1,030, broadly 1,560–1,610 cm^−1^),^29,30,33,35^ whereas Mock cells showed stronger nucleic acid signatures (725–735, 1,090–1,100, 1,330–1,340 cm^−1^).^20,29–31,33,36^ Additionally, we observed increased emphasis at 717–721 and 870–880 cm^−1^ for CD19-CAR T cells, consistent with membrane phosphatidylcholine (phospholipid) headgroup modes.^37^ Consistent with these protein and aromatic signature differences, single-cell RNA sequencing of CD19 CAR T cell infusion products from seven patients showed enrichment in CAR+ cells for proteostasis-associated pathways, including protein folding, as well as cell cycle processes (Supplementary Fig. 7c–d).

Together, this suggests that CAR expression shifts the probed cellular volume towards membrane, protein, and aromatic contributions, while Mock spectra show a greater dominance of nucleic acid bands. The increased protein and aromatic prominence in CD19-CAR T cells may reflect contributions from the CAR protein itself, which contains aromatic amino acids (tryptophan, tyrosine, and phenylalanine) in its scFv domain.^38^ Additionally, previous studies suggest that CAR proteins can self-assemble into raft-like domains stabilized by microvilli, which may locally concentrate membrane protein and phospholipid signatures, even in non-target-engaged cells.^39^ Conversely, Mock cells may produce spectra with relatively greater nuclear volume sampling, consistent with a more quiescent state and a less protein-rich membrane.

These trends were further validated using Raman spectra acquired without gold nanorods (~7,800 spectra, three donors, 25-second acquisitions; Methods), which remove surface-enhancement and substrate-specific biases. As expected, the adenine-associated bands were less pronounced in the absence of preferential gold nanorod binding, but the key class-dependent trends were preserved (Supplementary Fig. 8),^40^ supporting that these are genuine biochemical differences rather than SERS sampling bias.

Classification accuracy was further improved by isolating CD19-CAR positive cells from the ~4–45% untransduced fraction observed across all nine donors (reflecting typical lentiviral transduction efficiency) via fluorescence-activated cell sorting (FACS), followed by label-clearing step to minimize contributions from fluorescent antibody tags (Supplementary Fig. 9). Using models trained on the purified CAR T cells (~9,800 spectra across four donors), average donor-level classification accuracy increased from 70.3% to 80.6%, with all donors showing clear improvements in accuracy and area under the receiver operating characteristic curve (AUC) (Fig. 3d–e). This further demonstrates the detection sensitivity of this approach to CAR expression frequency.

### Time-resolved SERS of antigen-specific activation

**Fig. 4:**
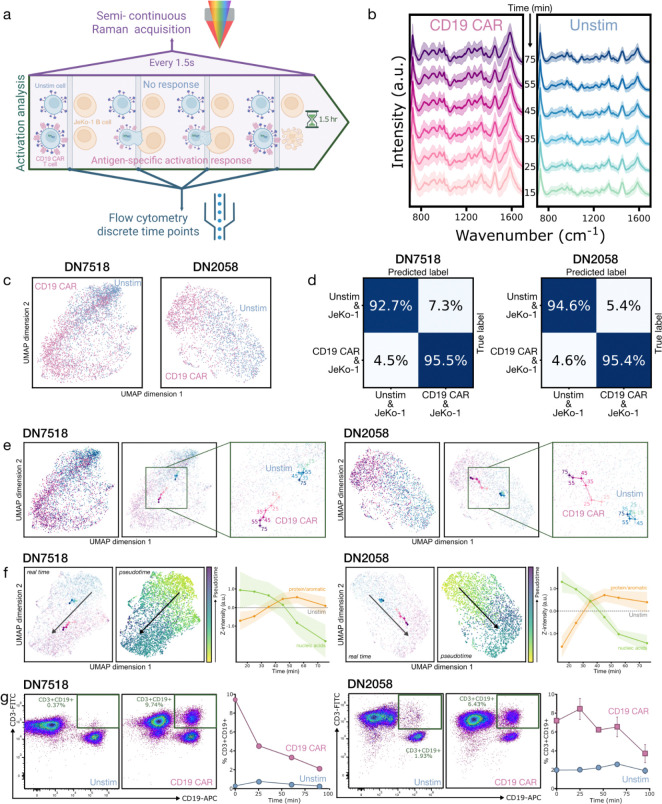
Antigen-specific activation analysis of CD19-CAR T cells by time-resolved SERS. **(a)** Diagram of experimental workflow. CD19-CAR and unstimulated T cells were co-cultured with CD19+ JeKo-1 B cells, where only CAR T cells undergo antigen-specific activation. Semi-continuous Raman acquisition was paired with discrete flow cytometry time points. **(b)** Mean normalized live-cell SERS spectra of CD19-CAR (pink) and unstimulated (blue) T cells co-cultured with JeKo-1 B cells, averaged in 10–20 minute segments (labels indicate segment start time from 15–75 minutes) over two donors. **(c)** Two-dimensional UMAPs of the Raman spectra plotted in (b). **(d)** Normalized confusion matrices displaying classification accuracy combined across the 1.5-hour time course for two donors. Accuracies were generated using a LightGBM classifier with 10-fold stratified cross-validation. **(e)** UMAPs of Raman spectra from two donors. Points are labeled by CAR (pink) and unstimulated (blue), with color shading from light to dark indicating time from 15 to 75 minutes (left). The centroids of each time segment are on the UMAP projection (middle). When zoomed in and labeled with their starting time point, they show a trajectory during activation (or lack thereof for unstimulated T cells) (right). **(f)** Pseudotime trajectory analysis of CAR T cell activation for two donors. UMAPs with real-time centroid trajectories, where darker hues indicate later time points, as in (e) (left). The pseudotime trajectories are visualized on UMAPs by a pseudotime color scale (yellow to purple) (middle). The arrows indicate the overall temporal direction. Co-cultured CAR T cell Raman peak intensity trends over real time for nucleic acid (green) and protein/aromatic (orange) bands, normalized to unstimulated cells (right). Curves represent median trajectories with shaded ribbons indicating 95% confidence intervals (n = 331–1,196 spectra/bin). **(g)** Flow cytometry validation of activation, measured by the CD3+CD19+ population (in the green box) for two donors. The flow fluorescence plots show representative distributions at the initial time point (marked as minute 0 on the right plot) (left, middle), and percentages of CD3+CD19+ cells are plotted over four discrete time points (right).

We next investigated whether label-free Raman spectroscopy could capture dynamic functional changes during antigen-specific activation. CD19-CAR and unstimulated (Unstim; isolated donor T cells without CD3/CD28 activation) T cells were co-cultured with CD19+ JeKo-1 B cells to induce antigen-specific activation of CAR T cells (Fig. 4a). Unstimulated T cells provided a baseline control for time-dependent spectral drift, ensuring we could isolate effects arising solely from activation and CAR expression. Cells were co-incubated at a 3:1 effector-to-target ratio that enriches for activation events.^41^ Spectra were acquired semi-continuously to monitor population-level activation dynamics (Fig. 4b; Methods). UMAP visualization of ~2,000 Raman spectra per donor across two donors revealed distinct clustering between CD19-CAR and Unstim conditions (Fig. 4c), with ML models achieving an average 94.6% classification accuracy via 10-fold stratified cross-validation (Fig. 4d).

Population-level comparisons aggregated across time (~7,600 spectra total) displayed differences reflecting both CD19-CAR expression and activation (Supplementary Fig. 10). Target-engaged CD19-CAR T cell spectra showed greater protein and aromatic prominence at 1,002 (phenylalanine), 1,031, and broadly 1,500–1,660 (amide I and II) cm^−1^,^29,30,32,33,35^ as well as increased signal in the 1220–1280 cm^−1^ (amide III) region. In contrast, Unstim T cells, in an even more quiescent state than Mock, had relatively enriched nucleic acid-associated signatures at 726 (adenine), 964, 1,095 (DNA backbone), 1,330–40, and 1,453 cm^−1^.^20,29–31,33,36^

These trends align with the CD19-CAR versus Mock differences we observed in individual cells — where SERS of protein-rich CAR T cells showed comparatively reduced nuclear sampling — and are also consistent with prior non-SERS Raman studies of T cell activation. The relative decrease in nucleic acid peaks during activation has been theorized to arise from chromatin decondensation, leading to a more open euchromatic architecture and therefore a lower local DNA concentration within the Raman probe volume.^20,21,29^ The broad increase in protein-related signatures for target-engaged CD19-CAR T cells is consistent with changes in protein composition and organization reported during T cell activation. During antigen-specific activation, T cells undergo synapse formation involving increased translation and protein synthesis, receptor recruitment to the membrane, cytoskeletal polarization, and membrane reorganization (including receptor aggregation).^29,39^ The co-cultured CD19-CAR T cell spectra showed increased relative signal within the 1220–1280 cm^−1^ region corresponding to amide III bands that reflect protein secondary structure and local environment and are sensitive to structural order.^33,35^ Increases in this band may indicate changes in protein backbone structure and bonding environment. Prior visible-excitation Raman studies have reported carotenoid-associated bands distinguishing T cells from B cells and decreasing with T cell activation.^20,31,42^ In our near-infrared (IR) SERS measurements, we did not observe prominent carotenoid contributions, consistent with much lower carotenoid Raman cross-sections in the near-IR^43^ and with potential additional effects from SERS sampling geometry.

Spectra from T- and JeKo-1 B-cell co-cultures captured the different stages of T-cell activation and B-cell cell death. To visualize activation progression, mean UMAP positions (“centroids”) for each condition were plotted over time (Fig. 4e). Although UMAP does not preserve global distances, qualitative shifts in the embedding can illustrate cluster evolution. For both donors, CD19-CAR centroids showed clear progression with time, indicating biochemical changes during activation, while Unstim centroids appeared comparatively stationary. As a complementary analysis, we applied an unsupervised pseudotime method that arranges spectra into a continuous trajectory based on their biochemical similarity (Fig. 4f, middle; Methods). This produced a pseudotime progression that strongly correlated with true experimental time (Spearman’s rank correlation of 1.00 and 0.94 for two donors; Fig. 4f, left; Supplementary Fig. 11).^44^ Notably, this method recovered an activation trajectory without using time labels, demonstrating that the Raman spectra inherently contain the temporal structure of activation.

Temporal tracking of spectral bands for both donors (Fig. 4f, right) displayed an initial rise in protein and aromatic bands during the first hour, followed by a partial decline (Spearman’s rank correlation of 0.83 and 0.6). Nucleic acid bands decreased monotonically, with a slight leveling after an hour (Spearman’s rank correlation of −1.00). The early trends are consistent with known activation processes mentioned above.^29,39^ Later leveling likely reflects a transition towards a steady effector state or population-level averaging over activated, interacting, and dying T and JeKo-1 B cells. We benchmark our findings against an established activation readout by performing parallel flow cytometry measurements of CD3+ CD19+ events — indicative of multi-cell events with interacting T and JeKo-1 B cells or trogocytosis.^38^ Activated CD19-CAR co-cultures showed a high early population of events that declined over time, while Unstim controls started low and remained unchanged (Fig. 4g; Supplementary Fig. 12). These measurements complement the Raman-derived trajectories, notably the later-time leveling, and provide an orthogonal validation of the activation dynamics captured with our label-free approach. Note that this flow assay detects conjugates, which may resolve more quickly, rather than underlying biochemical changes. Overall, these findings demonstrate that our approach enables dynamic live cell phenotyping with temporal resolution limited only by Raman acquisition time, in contrast to conventional flow cytometry, which captures fewer, more discrete time points and requires perturbative labeling and time-intensive processing.^21^

### Generalizability to a tonic signaling CAR (GD2-CAR)

**Fig. 5:**
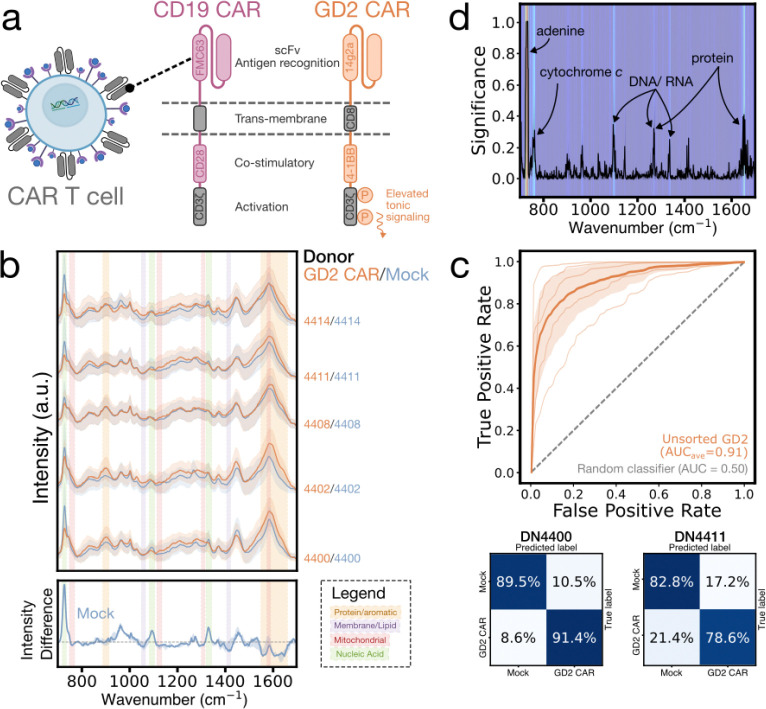
GD2-CAR and Mock T cell SERS classification. **(a)** Diagram comparing CD19-CAR (pink) and GD2-CAR (orange). Differences in antigen recognition and co-stimulatory domains are highlighted, with GD2-CAR showing elevated tonic signaling due to ζ-chain phosphorylation. **(b)** Waterfall plot of mean normalized SERS spectra of live single GD2-CAR (orange) and Mock (blue) T cells with ±1 SD (shaded), separated by donor (identifier labeled on the right) (top). Median donor-normalized spectral difference of Mock cells relative to CAR with 25–75% quantile range (bottom). The grey dashed line marks zero difference. Colored spectral bands highlight CAR-enriched protein/aromatic (gold), membrane/lipid (purple), and mitochondrial (red) regions, and Mock-enriched nucleic acid regions (green). **(c)** ROC curves displaying classification performance of GD2-CAR T cells versus Mock (top). Thick lines show the mean across four donors with ±1 SD and thin lines show individual donor curves. The grey dashed line represents a random classifier (AUC = 0.5). AUCs are given in the legend. Representative normalized confusion matrices for 2 of 5 donors displaying classification accuracy of Raman spectra using a LightGBM classifier with 10-fold stratified cross-validation (bottom). **(d)** Mean feature importance plot depicting the contribution of each spectral dimension (wavenumber) to classification, both through the curve and the heatmap background. Significant peaks are labeled with their biochemical vibrational modes.

To validate the robustness of this methodology across different CAR architectures, we generated and analyzed anti-GD2–4-1BB-CD3ζ CAR (GD2-CAR) T cells using the same activation, transduction, and expansion protocol as for CD19-CAR T cells. Donor-matched Mock T cells were generated in parallel and, as before, were activated and expanded but left untransduced. GD2-CAR T cells differ from CD19-CAR T cells in receptor antigen recognition and costimulatory domains (Fig. 5a). Additionally, GD2-CAR T cells exhibit high levels of tonic signaling, where clustering of the CAR surface proteins triggers basal ζ-chain phosphorylation and downstream metabolic activation independent of antigen recognition.^45^

Across five donors (~10,900 total spectra, Fig. 5b; Methods), donor-level classification accuracies averaged 85%, with average AUC improving from 0.78 (unsorted CD19-CAR) to 0.91 (Fig. 5c; Supplementary Fig. 13). Spectral analysis (Fig. 5b, bottom; Fig. 5d) showed that GD2-CAR T cells, like CD19-CAR T cells, displayed enriched protein and aromatic bands (892 and broadly around 1,600 cm^−1^)^30,33,35^ and weaker nucleic acid bands (728,^29–31^ 1,094, 1,335 cm^−1^)^20,29–31,33,36^. Additionally, GD2-CAR T cells have elevated mitochondrial signatures at 755, 1,130, 1,310, and 1,584 cm^−1^, consistent with cytochrome c-associated mitochondrial expansion.^20^ Orthogonal representative measurements — mitochondrial mass flow cytometry and mitochondrial fraction protein quantification — show higher mitochondrial content in GD2-CAR+ cells (Supplementary Fig. 14). These trends align with expected tonic signaling driven increases in protein synthesis, cellular stress, and mitochondrial content. Additionally, the 4–1BB costimulatory domain in the GD2-CAR variant favors fatty acid oxidation and mitochondrial biogenesis, resulting in elevated levels of cytochrome c complexes.^46^ Conversely, CD28 costimulation (found in the previous CD19-CAR construct) biases cells towards aerobic glycolysis (Warburg metabolism), which bypasses mitochondrial oxidation for energy production and leads to minimal mitochondrial expansion.^46,47^ This explains the lack of strong mitochondrial significance when comparing CD19-CAR and Mock T cell spectra. The higher classification accuracy for GD2-CAR demonstrates that as phenotypic changes increase, spectral separability strengthens. These results demonstrate that SERS can distinguish engineered T cell states driven by differences in a single membrane protein type, and can do so across distinct CAR architectures. This emphasizes the potential of our approach for profiling diverse engineered cell therapies.

## Discussion

We developed a label-free, SERS- and ML-based method for semi-continuous, non-destructive identification and functional state monitoring of live CAR T cells. Using a plasmonic platform, we collected rich molecular spectra that capture biochemical and biophysical changes resulting from blood cell type, engineered receptor expression, and activation processes. This demonstrates the value of this technique for probing not only cell identity, but also dynamic multidimensional functional state information with temporal specificity. In this work, we used a conservative 1.5-second acquisition time to ensure consistent high signal-to-noise across experimental conditions. This parameter can be readily reduced to the sub-second regime with speed-optimized configurations such as higher excitation power, greater plasmonic/nanophotonic enhancement, and improved optical throughput.^13,17^

Our spectral interpretations link vibrational molecular signatures to known CAR T cell biology, demonstrating the strength of Raman as a biochemistry-based readout. However, several challenges remain before achieving translational impact. Donor variability, transduction heterogeneity, and limited sample size currently hinder the generalizability of our model. Improving generalizability necessitates larger spectral libraries spanning multiple donors, constructs, and cell functional states. While SERS sampling bias and hotspotting can contribute to spectral variability, we have shown that complementary non-SERS measurements can help validate underlying biochemical features. Alternative SERS platforms, such as nanofabricated metasurfaces, have the potential to reduce sampling variability, improve reproducibility, and completely omit any cell-surface nanostructures. Our co-culture measurements inherently averaged across interacting and dying T and JeKo-1 B cells. Further studies tracking a single cell-cell interaction, either in microfluidics or an isolation well, would help remove fluctuations resulting from variations in cell state and type. Additional parallel biological assays could help deepen our spectral band interpretations (*e.g.,* information on metabolites, cytokines, or cell architecture).

Our approach’s rapid acquisition rate and minimal sample preparation make it well suited for future applications in quantifying proliferation kinetics, studying CAR-related toxicity, informing therapeutic decisions, and screening cells for functional phenotypes such as activation and, potentially, exhaustion. Engineered cells have transformative potential not only in cancer immunotherapy but also in autoimmune disease treatment. Both antitumor and immune-tolerizing engineered cell therapies (e.g., CAR T regulatory cells) would benefit from a unified approach to real-time monitoring of cell persistence, suppressive capacity, and functional stability. Beyond CD19- and GD2-CARs, the CAR protein makeup is subject to a large amount of variation. Future work can extend this approach across receptor constructs, including differences in intracellular costimulatory or signaling domains (e.g., CD3ε, CD4, CD8α) and extracellular CAR targets such as BCMA-CAR and CD22-CAR.^1^ Integrating this approach with microfluidic sample digitization could help create an automated, high-throughput point-of-care device for single-cell Raman profiling of engineered cells in blood.

To our knowledge, this work represents the first demonstration of Raman spectroscopy for single-cell phenotyping and dynamic functional monitoring of CAR T cells. Our approach is a novel, cross-disciplinary convergence of microscopy, nanophotonics, ML, biochemistry, and immunotherapy. As a rapid, non-destructive, and high-information-content technique, this method provides a foundation for gaining new insight into engineered cell mechanisms and helping ensure the development of safer and more effective CAR therapies.

## Methods

### Mammalian cell culture

The JeKo-1 human mantle cell lymphoma line (ATCC) was obtained as a gift from the Mackall Lab (Stanford, CA). Cells were cultured in complete growth medium — RPMI 1640 Medium with GlutaMAX supplement (Thermo Fisher Scientific), 10% heat-inactivated fetal bovine serum (FBS; R&D Systems), and 1% penicillin-streptomycin (10,000 U/mL; Gibco) — at 37°C, supplied with 5% CO_2_. Media was changed every two or three days. De-identified human red blood cells (K_2_ EDTA anticoagulant) were acquired from BioIVT and were stored at 4°C until use. De-identified primary human CD19+ CD20+ B cells were obtained cryopreserved from BioIVT and thawed day of measurement.

### CAR T cell generation and culture

De-identified human T cells were acquired through our institution’s blood bank and retrovirally transduced with CD19-CAR^25^ or GD2-CAR^3^ constructs as previously described. In brief, T cells were activated with the TransACT anti-CD3/anti-CD28 reagent (Miltenyi Biotech) and 100IU/mL rhIL-2 (PeproTech) for 48 h before retroviral transduction. On day 6 or 7, transduction efficiency was assessed via flow cytometry. Cells were labeled with huCD3 (BUV395; BD), huCD4 (BUV737; BD), huCD8 (Spark Blue 550; Biolegend), LIVE/DEAD fixable viability dye (Near IR; Invitrogen), and the respective CAR anti-idiotype conjugated to DyLight650 (as per manufacturer’s protocol; Invitrogen). Cells were cultured in complete culture medium supplemented with 10 ng/mL rhIL-2 at 37°C with 5% CO_2_, with media changes every two or three days.

### Nanorod synthesis and characterization

Gold nanorods were prepared using a seed-mediated growth method adapted from a previously reported protocol.^27^ All chemicals were obtained from Sigma-Aldrich and used without further purification.

The seed solution for gold nanorod growth was prepared as follows: 5 mL of 0.5 mM HauCl_4_ was combined with 5 mL of 0.2 M cetyltrimethylammonium bromide (CTAB) in a 20 mL vial. Fresh 0.01 M NaBH_4_ (0.6 mL, diluted to 1 mL with water) was rapidly injected into the Au(III)-CTAB solution under vigorous stirring at 1,200 rpm. Stirring was stopped after 2 min, and the seed solution was aged at room temperature for 30 min.

For the growth solution, CTAB (43 mM) and NaOL (9 mM) were dissolved in 475 mL of water, followed by the addition of 12 mL of 4 mM AgNO₃ and 10 mL of 25 mM HAuCl_4_. The pH was adjusted with 12 M HCl (1.5 mL or 2.1 mL for the two nanorod batches), and the solution was reduced using 0.8 mL of 100 mM ascorbic acid. The seed solution was then added to the growth solution at 30 °C and left undisturbed for 12 h to yield Au nanorods. Nanorods were collected by centrifugation and washed twice. As-synthesized nanorods were stabilized with 5 mM CTAB.

Washed nanorod resonance wavelengths were determined via ultraviolet-visible spectroscopy (Cary 6000i UV-Vis-NIR; Agilent Technologies). Size distributions were measured manually in FIJI, image analysis software, using transmission electron microscope images acquired at 200kV (Tecnai G2 F20 X-TWIN; Thermo Fisher Scientific).^48^ Gold concentrations were quantified by inductively coupled plasma optical emission spectroscopy (Thermo iCAP 6300; Thermo Fisher Scientific). Two nanorod batches were used in this study (Supplementary Fig. 1). Nanorods used for donors DN71, DN74, and DN76 exhibited a longitudinal resonance at 686 nm, with an aspect ratio of 2.73 ± 0.66 and a gold concentration of 420 μg/mL. Nanorods used for all other donors exhibited a resonance at 678 nm with an aspect ratio of 2.13 ± 0.78, and gold concentration of 350 μg/mL.

### Cell and nanorod sample preparation

Spectra were collected from T cells on day 13 or 14 post-CD3/CD28 activation. For JeKo-1 cells, measurements were collected 3–4 days after thawing. For primary B cells, measurements were collected on the day of thawing. Cells were washed three times in PBS and counted using an automated cell counter (LUNA-BX7^™^; Logos Biosystems). For SERS measurements, cells were mixed 1:1 by volume with gold nanorods (omitted for non-SERS controls) to generate final cell concentrations of approximately 6 × 10^4^ cells/μL (primary B and T cells), 2.1 × 10^4^ cells/μL (JeKo-1 B cells), and 1.5 × 10^5^ cells/μL (RBCs), sufficient to form an approximately single-cell layer at the base of the well. For co-culturing experiments, T cells and CD19-positive JeKo-1 B cells were mixed at a 3:1 effector-to-target ratio, common for *in vitro* assays that enrich for activation events.^41^ This is elevated from the physiological ratio observed *in vivo*, but allowed us to capture a high population of activated cells in our excitation spot.^49^

CTAB-stabilized nanorods were sonicated, then washed twice by centrifugation in water to remove excess cytotoxic surfactant^50^ while maintaining colloidal stability. After the final wash, nanorods were resuspended in half the original volume by sonication and mixed 1:1 with cells, restoring to the nominal stock concentration. Because washing introduces loss through aggregation and adhesion, the effective concentration is likely lower than nominal, but our protocol was kept consistent across all experiments.

### Liquid well preparation

Liquid wells were fabricated on 25 × 25 × 1 mm glass chips. Slides were plasma cleaned for 5 minutes at 100 W (PX-250 Plasma Asher; March Instruments; 85 mTorr, 2 SCCM O_2_, direct) and coated with a 5 nm Ti adhesion layer followed by 195 nm Au to provide reflectivity and low Raman background. Liquid wells were assembled following a method adapted from previous work.^51^ In brief, two layers of double-sided tape were hole-punched to create a spacer and applied to the gold-coated surface. The inner edges of the hole were coated in high-vacuum grease (Dow Corning) to prevent leakage. A 4 μL sample was loaded into the well and sealed with a borosilicate glass coverslip (Corning) to prevent sample evaporation during Raman acquisition.

### Flow cytometry / Fluorescence-Activated Cell Sorting

For activation analysis studies, CD19-CAR T cells, Mock T cells, and JeKo-1 B cells were washed by centrifugation and resuspended in phosphate buffered saline (PBS; Gibco). T cells were co-cultured with JeKo-1 B cells at a 3:1 effector-to-target ratio in PBS. For each donor, samples were collected at predefined timepoints (DN7518: 0, 25, 60, 90 min; DN2058 (in duplicate): 0, 25, 45, 65, 90 min). At each time point, an aliquot of ~10^5^ cells was stained on ice with huCD3 (FITC; BioLegend), huCD19 (APC; BioLegend) at a 1:200 dilution, fixed (Fixation Buffer; Invitrogen), washed, and resuspended in flow buffer (PBS, 2% FBS, and 1 mM EDTA; Invitrogen). Single-color and unstained controls were used for compensation and gating (gating and all density plots are shown in Supplementary Fig. 12). Flow cytometry was performed on an ACEA Novocyte Quanteon (Agilent), and activation was tracked as CD3+CD19+ events using FlowJo software (BD Biosciences).^52^

For experiments using purified CD19-CAR T cells, DyLight 650-conjugated FMC63 anti-idiotype was used to label CD19-CAR T cells, which were then bulk sorted using a FACSAria II (BD Biosciences). Sorted cells were incubated overnight at 37°C and washed three times in PBS to allow fluorescent label removal before Raman analysis.

For mitochondrial mass measurements, donor-matched FMC-CAR, GD2-CAR, and Mock T cells were stained at room temperature for 20 minutes in the dark with a DyLight 650-conjugated anti-idiotype antibody to identify CAR+ and CAR− populations and with 2 μg/mL nonyl acridine orange (NAO; Invitrogen) to assess mitochondrial mass. Flow cytometry was performed on a Cytek Aurora and analysis was performed in FlowJo. Cells were gated for lymphocytes, singlets, and CAR expression (Supplementary Fig. 14a–c). NAO fluorescence was quantified as the median fluorescence intensity within the CAR+ and CAR− gates for CAR-transduced samples and across the full Mock population (Supplementary Fig. 14d–e).

### Mitochondrial extraction

Mitochondria were extracted from T cells using a mitochondria isolation kit for cultured cells (Thermo Scientific) according to the manufacturer’s protocol to obtain cytosolic and mitochondrial fractions. Mitochondrial pellets were lysed in 2% CHAPS (Abcam) in Tris-buffered saline. Mitochondrial-fraction protein concentration was quantified using a Bradford assay (Bio-Rad) by measuring absorbance at 595 nm on a spectrophotometer (Bio-Rad). For CAR-transduced samples, concentrations were adjusted by transduction efficiency to provide an estimated CAR+-adjusted mitochondrial protein concentraiton (assuming the non-transduced component resembles Mock).

### Raman spectroscopy

Raman spectra were collected on an XploRA+ confocal Raman microscope (HORIBA Scientific) equipped with a 1,200 gr/mm grating, 100 μm slit, 300 μm pinhole, and 785 nm diode excitation laser. Spectra were acquired over the 700–1,700 cm^−1^ biological fingerprint region on a rectangular scan grid with ~30 μm spacing, exceeding both the laser spot size (~1.2–1.5 μm) and typical cell diameters (7–12 μm), ensuring approximately single-cell sampling. All SERS measurements were taken with 1.5-second acquisition times.

Non-enhanced spectra (Supplementary Fig. 8) were obtained with 25-second acquisitions. Because of laser-induced cell drift (likely due to local heating) during longer exposures, spectra were collected in small batches (10–15 spectra) with the excitation location selected via point-by-point to remain centered on single cells.

Measurements for Figs. 3 and 5 were performed using a 50x/0.65 NA objective (Olympus LCPLAN 50X IR; Evident Scientific) with the correction collar set to 0.5. Under 785 nm excitation, this results in a calculated diffraction-limited spot size of ~1.5 μm. Co-cultured activation and immune cell type measurements (Fig. 2 and 4) were taken using a 20x/0.80 NA objective (Olympus UPlanXApo; Evident Scientific), with a theoretical spot size of ~1.2 μm. Actual spot sizes were larger due to sample-induced aberrations and scattering in liquid conditions.

All spectra were acquired with 100% power output: 30.47 mW at the sample for the 20x objective and 26.34 mW for the 50x. Before acquisition, samples were allowed to settle for 15 minutes (co-culturing experiments) or 20 minutes (for single-cell experiments).

### Cell viability

CAR T cells were loaded into liquid wells described above, where tape is coated with high-vacuum grease to allow repeated opening for sample access. Cell viability was assessed using acridine orange/propidium iodide staining (Logos Biosystems) and quantified on an automated cell counter. Cells were incubated for 1 hour at room temperature under four conditions: with gold nanorods, without gold nanorods, with continuous 785 nm Raman excitation, and without laser exposure. Controls were also obtained at 0 minutes, with and without nanorod addition. Nanorod concentrations and Raman excitation were conducted in the same conditions used for SERS experiments. Each condition was performed in duplicate using independently prepared cell and nanorod samples.

### Scanning electron microscopy (SEM)

Cells were fixed in 4% paraformaldehyde (Sigma-Aldrich) and 0.1% glutaraldehyde (Sigma-Aldrich) in PBS for 30 min, followed by three washes to remove salts (one wash in PBS and two in water, with 5 min equilibration between spins). Fixed cells were mixed with nanorods and 2 μL droplets of the suspension were air dried on silicon chips. Samples were sputter-coated with ~5 nm of Au/Pd alloy (60/40 wt%; Cressington 108 Auto Sputter Coater; Cressington Scientific Instruments). Imaging was performed on a FEI Magellan 400 XHR scanning electron microscope (Thermo Fisher Scientific) using the concentric backscattered detector at 5 kV landing voltage, 50 pA beam current, and −3 kV stage bias.

### Cryo-electron microscopy and tomography (cryo-EM/ET)

300 mesh molybdenum lacey carbon grids (LC300-MO; Electron Microscopy Sciences) were glow discharged for 10 seconds at 10 mA (PELCO easiGlow^™^ Glow Discharge Cleaning System; Ted Pella, Inc.). Cell-nanorod mixtures (3μL) were applied to each grid, blotted for 3 seconds, and vitrified in liquid ethane using a Vitrobot^™^ Mark IV (Thermo Fisher Scientific). Grids were imaged on a Glacios^™^ 200kV cryo-transmission electron microscope using Tomography 5 (Thermo Fisher Scientific).

Tomographic tilt series were acquired from −60° to +60°. Tilt images were aligned and reconstructed using IMOD (Etomo interface).^53^ Reconstructed tomograms were visualized in UCSF ChimeraX^54^.

### ζ-potential characterization

Cell and nanorod ζ-potential values were measured in a 1:1 mixture of PBS and water to match experimental conditions using Phase Analysis Light Scattering (Nanobrook Omni; Brookhaven Instruments). ζ-potential measurements were performed for mammalian immune cells, red blood cells, and bacterial reference strains to benchmark relative surface charges. Each sample was measured in triplicate, with 30 cycles per measurement. Red blood cells, which have more heavily sialylated membrane glycoproteins, exhibited a more negative ζ-potential (~ −17 mV) than white blood cells (~ −12 mV).^55^ This was consistent with the greater nanorod binding observed in dried SEM samples (Fig. 2e–f).

### Single-cell RNA sequencing analysis

Single-cell RNA sequencing (scRNA-seq) was performed on CD19 CAR T cell infusion products from seven patients with large B-cell lymphoma (LBCL) treated with axicabtagene ciloleucel (axi-cel; Yescarta, Kite Pharma) as standard-of-care therapy at our institution. Patients provided informed consent through Clinical Outcomes Biorepository (Stanford IRB #43375), and clinical metadata were obtained via retrospective chart review.

Raw sequencing data were processed and analyzed using Seurat v5.0.^56^ Cells were filtered to remove ones with greater than 10% mitochondrial gene content, as well as low-quality cells based on total UMI counts and number of detected genes. After quality control, the final dataset comprised 102,359 cells across 36,604 genes.

To identify transcriptional differences between CD19 CAR-expressing (CAR+; n = 82,552) and non-expressing (CAR−; n = 19,807) cells, differential expression analysis was performed using Seurat’s FindMarkers pipeline. Only genes expressed in ≥5% of cells in either group were retained. TCR and BCR clonotypes, as well as sex-associated genes, were removed prior to testing. The Wilcoxon rank-sum test was used as the default statistical method to identify differentially expressed genes (DEGs) and differential regulon activity and results were visualized using the EnhancedVolcano package.

Transcription factor network (regulon) activity was inferred using SCENIC (Single-Cell rEgulatory Network Inference and Clustering).^57^ Regulons were scored per cell using the AUCell algorithm,^58^ and differential regulon activity between CAR+ and CAR− cells was assessed using the Wilcoxon rank-sum test. Pathway enrichment analysis was performed on differential expression analysis. Adjusted p-values were computed using the Benjamini–Hochberg procedure to control the false discovery rate.

### Data preprocessing

All preprocessing and computational analyses were performed in Jupyter notebooks (Python 3.11.13) and executed on a CPU.

Raman spectra were preprocessed following a method adapted from previous work that included despiking, denoising, baseline correction, and normalization (Supplementary Fig. 4).^23^ Cosmic ray spikes were removed using a modified Whitaker-Hayes z-score method.^59^ Despiked spectra were denoised by wavelet thresholding (BayesShrink; skimage) to suppress high-frequency noise while preserving Raman peaks.^60^ Baseline drift was corrected using adaptive iteratively reweighted penalized least squares (airPLS).^61^ Finally, spectra were z-score standardized on a per-spectrum basis for cross-sample comparisons and downstream analyses. For each acquisition period, spectra with average unnormalized intensities exceeding three standard deviations above that experiment’s mean were excluded to avoid laser-induced artifacts or detector saturation.

### Spectral plotting and intensity analyses

To uncover spectral differences between conditions, we computed per-donor intensity difference curves by subtracting class-averaged spectra. Difference curves were normalized to range −1 to 1, then median-aggregated across donors and plotted with 95% confidence bands. To analyze time-resolved activation dynamics, spectra were grouped into time bins labeled by their earliest time point (15–25, 25–35, 35–45, 45–55, 55–75, and 75–95 min). For plotting, mean spectra were computed within each time bin. Temporal changes were tracked in 20 wavenumber bands centered on protein and aromatic signatures (898, 1,002, 1,025, 1,222, 1,262, 1,602 cm^−1^)^30,33,35^ and nucleic acid signatures (728, 1,092, 1,334 cm^−1^).^20,29–31,33,36^ For activation analysis, spectral intensities in target-engaged CAR T cell co-cultures were computed relative to Unstim controls using log-ratio differences to remove global acquisition-related variation with time. Within each biochemical class, intensities were z-score standardized within bands and averaged across them. Confidence intervals were estimated using block-bootstrap resampling. Monotonicity of trends was assessed using two-sided Spearman’s rank correlation between z-scored intensities and experimental time (scipy).

### Dimensionality reduction and manifold projection

Preprocessed spectra were visualized using Uniform Manifold Approximation and Projection (UMAP; n_components = 2, n_neighbors = 20, min_dist = 0.1, metric = “manhattan”).^28^ UMAPs were generated using spectra balanced by donor and class to avoid overweighting. For qualitative illustration of activation dynamics, we computed the centroid of each class within each time bin by calculating the mean UMAP coordinate. Uncertainty in centroid location was estimated using the standard error of the coordinates.

### Machine learning classification and feature importance

Lightweight ensemble Light Gradient Boosting Machine (LightGBM) models were trained for spectral classification.^62^ LightGBM demonstrated the best trade-off between accuracy and computational efficiency in our application among the ensemble models evaluated (Supplementary Table 3). All hyperparameter and overfitting tuning were performed on the training data. First- and second-order Savitzky-Golay derivatives (window_length = 11, polyorder = 3; scipy) were appended as additional dimensions so that slope and curvature information could also inform classification accuracy.^63^ Derivative-augmented data were used for all classification analysis; non-augmented spectra were used for feature-importance analysis for interpretability.

Prior to model training, datasets were split into 80:20 train:test sets, stratified by class. Hyperparameters were optimized via Bayesian optimization (Optuna)^64^ using five-fold cross-validation (CV) stratified by class, with early stopping based on validation performance (training terminated if AUC didn’t improve for 150 consecutive boosting rounds), and a maximum of 2,000 boosting rounds. The scale_pos_weight parameter was adjusted based on class imbalance. Learning curves from five-fold CV were used to estimate the optimal number of boosted trees (n_estimators) that avoided overfitting while maximizing validation AUC. Final models were retrained and evaluated using 10-fold CV stratified by class on the training data, and performance was assessed through confusion matrices (scikit-learn). Receiver operating characteristic (ROC) curves and their AUC values were computed on held-out test sets (scikit-learn). For per-donor models, ROC curves were interpolated onto a common grid, bootstrap averaged, and plotted with 95% confidence intervals.

For immune cell type classification, a multiclass dataset, the same workflow was used except with log loss minimized rather than AUC maximized. For CAR vs Mock T cell classification, when models were trained across all donors, pooled accuracy reached 69.3% for CD19-CAR versus Mock and 81.4% for GD2-CAR versus Mock. However, UMAP visualization showed that inter-donor variability exceeded between-class differences (Supplementary Fig. 6a), indicating a strong donor prior and motivating a donor-level training scheme, given the available dataset sizes.^65^

To identify spectral features that best improved our model’s accuracy, we used LightGBM’s intrinsic gain-based feature importance scores for models trained on non-derivative-augmented spectra.^62^ Per-donor importance profiles were sum-normalized to account for magnitude variation and scaled from 0 to 1. Mean gain profiles were then computed, and uncertainty was estimated using the standard deviation across donors.

### Pseudotime analysis

Pseudotime trajectories were generated from spectra projected into a 45-dimensional PCA space (scikit-learn). We computed a minimum spanning tree, rooted at the earliest time point cell, on the largest connected component of a k-nearest-neighbor graph (k-NN, k = 30 for DN2058, k = 40 for DN7518; cosine distance). For trajectory visualization (Supplementary Fig. 11), pseudotime values were binned into 12 clusters, and the centroid of each cluster was overlaid onto a UMAP of PCA-reduced data. Correlation between pseudotime and experimental time was evaluated using two-sided Spearman’s rank correlation (scipy).

### Data size and handling

The number of independent single-cell Raman spectra collected for each donor and condition is reported in Supplementary Table 2. Across experiments, sample sizes ranged from 900 to 5840 spectra per condition and donor. Each spectrum corresponds to a distinct single cell; no repeated measurements of the same cell were taken. All analyses were performed either per-donor and/or using stratified cross-validation with balanced donors. No statistical models including covariates were used.

## Supplementary Material

Supplement 1

Supplement 2 **Supplementary Video 1:** Aligned cryo-ET tilt series of a whole T cell with gold nanorods Aligned cryo-electron tomography (cryo-ET) tilt series of a T cell with gold nanorods deposited on a lacey carbon grid (corresponding in Supplementary Fig. 2a). Smaller high-contrast features represent nanorods present on the cell surface and grid.

Supplement 3 **Supplementary Video 2:** Reconstructed tomogram of the tilt series shown in Supplementary Video 1
Three-dimensional tomogram reconstructed from the aligned tilt series shown in Supplementary Video 1. High-intensity features correspond to nanorods.

Supplement 4 **Supplementary Video 3:** Aligned cryo-ET tilt series of two T cells with gold nanorods Aligned cryo-electron tomography (cryo-ET) tilt series of two T cells with gold nanorods deposited on a lacey carbon grid (corresponding in Supplementary Fig. 2b). Smaller high-contrast features represent nanorods present on the cell surface and grid.

Additional Information

Supplementary Information is available for this paper.

## Data Availability

The source data that support the findings in this work are available from the corresponding authors upon reasonable request.

## Code Availability

The code used to analyze and classify Raman spectral data relies on pre-built Python packages and is available from the corresponding authors upon reasonable request.

## References

[1] Labanieh L. & Mackall C. L. CAR immune cells: design principles, resistance and the next generation. Nature 614, 635–648 (2023). 36813894 10.1038/s41586-023-05707-3

[2] Locke Frederick L. Axicabtagene Ciloleucel as Second-Line Therapy for Large B-Cell Lymphoma. N. Engl. J. Med. 386, 640–654 (2022). 34891224 10.1056/NEJMoa2116133

[3] Mount C. W. Potent antitumor efficacy of anti-GD2 CAR T cells in H3-K27M+ diffuse midline gliomas. Nat. Med. 24, 572–579 (2018). 29662203 10.1038/s41591-018-0006-x PMC6214371

[4] Grupp S. A. Chimeric antigen receptor–modified T cells for acute lymphoid leukemia. N. Engl. J. Med. 368, 1509–1518 (2013). 23527958 10.1056/NEJMoa1215134 PMC4058440

[5] Locke F. L. Long-term safety and activity of axicabtagene ciloleucel in refractory large B-cell lymphoma (ZUMA-1): a single-arm, multicentre, phase 1–2 trial. Lancet Oncol. 20, 31–42 (2019). 30518502 10.1016/S1470-2045(18)30864-7 PMC6733402

[6] Baysoy A., Bai Z., Satija R. & Fan R. The technological landscape and applications of single-cell multi-omics. Nat. Rev. Mol. Cell Biol. 24, 695–713 (2023). 37280296 10.1038/s41580-023-00615-w PMC10242609

[7] Hu Y. & Huang J. The chimeric antigen receptor detection toolkit. Front. Immunol. 11, 1770 (2020). 32849635 10.3389/fimmu.2020.01770 PMC7431616

[8] Andrzejewska A. Labeling of human mesenchymal stem cells with different classes of vital stains: robustness and toxicity. Stem Cell Res. Ther. 10, 187 (2019). 31238982 10.1186/s13287-019-1296-8 PMC6593614

[9] Teranishi K. Label-free ghost cytometry for manufacturing of cell therapy products. Sci. Rep. 14, 21848 (2024). 39300150 10.1038/s41598-024-72016-8 PMC11413197

[10] Jain M. D. & Spiegel J. Y. Imagining the cell therapist: Future CAR T cell monitoring and intervention strategies to improve patient outcomes. EJHaem 3, 46–53 (2022). 35844298 10.1002/jha2.357 PMC9175904

[11] Salek M. COSMOS: a platform for real-time morphology-based, label-free cell sorting using deep learning. Commun. Biol. 6, 971 (2023). 37740030 10.1038/s42003-023-05325-9 PMC10516940

[12] Zhang Y. From genotype to phenotype: Raman spectroscopy and machine learning for label-free single-cell analysis. ACS Nano 18, 18101–18117 (2024). 38950145 10.1021/acsnano.4c04282

[13] Safir F. Combining Acoustic Bioprinting with AI-Assisted Raman Spectroscopy for High-Throughput Identification of Bacteria in Blood. Nano Lett. 23, 2065–2073 (2023). 36856600 10.1021/acs.nanolett.2c03015 PMC10037319

[14] Wang X., Huang S.-C., Hu S., Yan S. & Ren B. Fundamental understanding and applications of plasmon-enhanced Raman spectroscopy. Nature Reviews Physics 2, 253–271 (2020).

[15] Zhang R. Chemical mapping of a single molecule by plasmon-enhanced Raman scattering. Nature 498, 82–86 (2013). 23739426 10.1038/nature12151

[16] Tadesse L. F. Plasmonic and Electrostatic Interactions Enable Uniformly Enhanced Liquid Bacterial Surface-Enhanced Raman Scattering (SERS). Nano Lett. 20, 7655–7661 (2020). 32914987 10.1021/acs.nanolett.0c03189 PMC7564787

[17] Schie I. W. High-throughput screening Raman spectroscopy platform for label-free Cellomics. Anal. Chem. 90, 2023–2030 (2018). 29286634 10.1021/acs.analchem.7b04127

[18] Ichimura T. Non-label immune cell state prediction using Raman spectroscopy. Sci. Rep. 6, 37562 (2016). 27876845 10.1038/srep37562 PMC5120326

[19] Akagi Y., Mori N., Kawamura T., Takayama Y. & Kida Y. S. Non-invasive cell classification using the Paint Raman Express Spectroscopy System (PRESS). Sci. Rep. 11, 8818 (2021). 33893362 10.1038/s41598-021-88056-3 PMC8065115

[20] Borek-Dorosz A. Alterations in lipid metabolism accompanied by changes in protein and carotenoid content as spectroscopic markers of human T cell activation. Biochim. Biophys. Acta Mol. Cell Biol. Lipids 1869, 159496 (2024). 38649008 10.1016/j.bbalip.2024.159496

[21] Pavillon N. & Smith N. I. Non-invasive monitoring of T cell differentiation through Raman spectroscopy. Sci. Rep. 13, 3129 (2023). 36813799 10.1038/s41598-023-29259-8 PMC9947172

[22] Kobayashi-Kirschvink K. J. Prediction of single-cell RNA expression profiles in live cells by Raman microscopy with Raman2RNA. Nat. Biotechnol. (2024) doi:10.1038/s41587-023-02082-2. PMC11233426 38200118

[23] Chang K. Predicting targeted- and immunotherapeutic response outcomes in melanoma with single-cell Raman Spectroscopy and AI. Cancer Biology (2025). 10.1200/PO-25-00591 42715507

[24] Shipp D. W., Sinjab F. & Notingher I. Raman spectroscopy: techniques and applications in the life sciences. Adv. Opt. Photon., AOP doi:10.1364/AOP.9.000315.

[25] Kochenderfer J. N. Construction and preclinical evaluation of an anti-CD19 chimeric antigen receptor. J. Immunother. 32, 689–702 (2009). 19561539 10.1097/CJI.0b013e3181ac6138 PMC2747302

[26] Dan L. & Kang-Zheng L. Optimizing viral transduction in immune cell therapy manufacturing: key process design considerations. J. Transl. Med. 23, 501 (2025). 40316943 10.1186/s12967-025-06524-0 PMC12046913

[27] Ye X., Zheng C., Chen J., Gao Y. & Murray C. B. Using binary surfactant mixtures to simultaneously improve the dimensional tunability and monodispersity in the seeded growth of gold nanorods. Nano Lett. 13, 765–771 (2013). 23286198 10.1021/nl304478h

[28] McInnes L., Healy J., Saul N. & Großberger L. UMAP: Uniform Manifold Approximation and Projection. J. Open Source Softw. 3, 861 (2018).

[29] Chaudhary N., Nguyen T. N. Q., Cullen D., Meade A. D. & Wynne C. Discrimination of immune cell activation using Raman micro-spectroscopy in an in-vitro & ex-vivo model. Spectrochim. Acta A Mol. Biomol. Spectrosc. 248, 119118 (2021). 33214105 10.1016/j.saa.2020.119118

[30] Chan J. W. Micro-Raman spectroscopy detects individual neoplastic and normal hematopoietic cells. Biophys. J. 90, 648–656 (2006). 16239327 10.1529/biophysj.105.066761 PMC1367069

[31] Borek-Dorosz A. Raman-based spectrophenotyping of the most important cells of the immune system. J. Adv. Res. 41, 191–203 (2022). 36328748 10.1016/j.jare.2021.12.013 PMC9637483

[32] Premasiri W. R., Lee J. C. & Ziegler L. D. Surface-enhanced Raman scattering of whole human blood, blood plasma, and red blood cells: cellular processes and bioanalytical sensing. J. Phys. Chem. B 116, 9376–9386 (2012). 22780445 10.1021/jp304932g PMC3704210

[33] Parker F. S. Biochemical applications of infrared and Raman spectroscopy. Appl. Spectrosc. 29, 129–147 (1975).

[34] Atkins C. G., Buckley K., Blades M. W. & Turner R. F. B. Raman spectroscopy of blood and blood components. Appl. Spectrosc. 71, 767–793 (2017). 28398071 10.1177/0003702816686593

[35] Stepanenko T. Surface-enhanced Raman scattering (SERS) and tip-enhanced Raman scattering (TERS) in label-free characterization of erythrocyte membranes and extracellular vesicles at the nano-scale and molecular level. Analyst 149, 778–788 (2024). 38109075 10.1039/d3an01658g

[36] Safar W., Azziz A., Edely M. & Lamy de la Chapelle M. Conventional Raman, SERS and TERS studies of DNA compounds. Chemosensors (Basel) 11, 399 (2023).

[37] Akutsu H. Structure and dynamics of phospholipids in membranes elucidated by combined use of NMR and vibrational spectroscopies. Biochim. Biophys. Acta Biomembr. 1862, 183352 (2020). 32407775 10.1016/j.bbamem.2020.183352

[38] He C. CD19 CAR antigen engagement mechanisms and affinity tuning. Sci. Immunol. 8, eadf1426 (2023). 36867678 10.1126/sciimmunol.adf1426 PMC10228544

[39] Beppler C. Hyperstabilization of T cell microvilli contacts by chimeric antigen receptors. J. Cell Biol. 222, (2023). 10.1083/jcb.202205118 PMC9757849 36520493

[40] Barhoumi A. & Halas N. J. Label-free detection of DNA hybridization using surface enhanced Raman spectroscopy. J. Am. Chem. Soc. 132, 12792–12793 (2010). 20738091 10.1021/ja105678z

[41] Bandara V. Pre-clinical validation of a pan-cancer CAR-T cell immunotherapy targeting nfP2X7. Nat. Commun. 14, 5546 (2023). 37684239 10.1038/s41467-023-41338-y PMC10491676

[42] Laskowska P. Raman spectroscopy imaging in evaluation of immune cells activation status. Blood 142, 7157–7157 (2023).

[43] Udensi J., Loughman J., Loskutova E. & Byrne H. J. Raman spectroscopy of carotenoid compounds for clinical applications-A review. Molecules 27, 9017 (2022). 36558154 10.3390/molecules27249017 PMC9784873

[44] Saelens W., Cannoodt R., Todorov H. & Saeys Y. A comparison of single-cell trajectory inference methods. Nat. Biotechnol. 37, 547–554 (2019). 30936559 10.1038/s41587-019-0071-9

[45] Chen J. Tuning charge density of chimeric antigen receptor optimizes tonic signaling and CAR-T cell fitness. Cell Res. 33, 341–354 (2023). 36882513 10.1038/s41422-023-00789-0 PMC10156745

[46] Kawalekar O. U. Distinct signaling of coreceptors regulates specific metabolism pathways and impacts memory development in CAR T cells. Immunity 44, 380–390 (2016). 26885860 10.1016/j.immuni.2016.01.021 PMC13498396

[47] Pearce E. L. & Pearce E. J. Metabolic pathways in immune cell activation and quiescence. Immunity 38, 633–643 (2013). 23601682 10.1016/j.immuni.2013.04.005 PMC3654249

