# Supplementary material for: Dynamic, single-cell monitoring of CAR T cell identity and activation with Raman spectroscopy
Source: bioRxiv. 2026 Feb 23:2026.02.22.707331. Preprint. [Version 1] doi: 10.64898/2026.02.22.707331 (PMC13160088; doi:10.64898/2026.02.22.707331)
Supplement: Supplement 1 [file media-1.pdf]

# Supplementary Information: Dynamic, single-cell monitoring of CAR T cell identity and activation with Raman spectroscopy

Ariel Stiber[1*], Boi Quach[2-4], Babatunde Ogunlade[1], Antony Georgiadis[1], Kai Chang[5], Yuanwei Li[1], Patrick Quinn[4], Haoqing Wang[6,7], Kristin C. Y. Tsui[2-4], Charm Ang[1], Elena Sotillo[4,11], Crystal Mackall[4,8-11*], Zinaida Good[2-4,10,11*], Jennifer A. Dionne[1,12*]

[1] Department of Materials Science and Engineering, Stanford University, Stanford, California, USA

[2] Division of Immunology and Rheumatology, Department of Medicine, Stanford University School of Medicine, Stanford, California, USA

[3] Division of Computational Medicine, Department of Medicine, Stanford University School of Medicine, Stanford, California, USA

[4] Center for Cancer Cell Therapy, Stanford Cancer Institute, Stanford University, Stanford, California, USA

[5] Department of Electrical Engineering, Stanford University, Stanford, California, USA

[6] Stanford Cryo-EM Microscopy Center, Stanford University, Stanford, California, USA

[7] Cryoelectron Microscopy, Nucleus at Sarafan ChEM-H, Stanford University, Stanford, California, USA

[8] Division of Hematology and Oncology, Department of Pediatrics, Stanford University, Stanford, CA, USA

[9] Division of Blood and Marrow Transplantation and Cellular Therapy, Department of Medicine, Stanford University, Stanford, California, USA

[10] Parker Institute for Cancer Immunotherapy, Stanford University, Stanford, California, USA

[11] Weill Cancer Hub West, Stanford University, Stanford, California, USA

[12] Department of Radiology, Stanford University School of Medicine, Stanford, California, USA

*Corresponding authors. Emails: astiber@stanford.edu; cmackall@stanford.edu; zinaida@stanford.edu; jdionne@stanford.edu

# Table of Contents

SUPPLEMENTARY TABLE 1: TRANSDUCTION EFFICIENCIES FOR ALL DONORS....................................... 3
SUPPLEMENTARY FIG. 1: GOLD NANOROD CHARACTERIZATION................................................................ 4
SUPPLEMENTARY FIG. 2: CRYO-ELECTRON MICROGRAPHS OF T CELLS WITH NANORODS................. 5
SUPPLEMENTARY FIG. 3: CELLULAR SERS VS. NANOROD AND NON-SERS CONTROLS..........................6
SUPPLEMENTARY TABLE 2: SPECTRAL COUNT FOR EACH SAMPLE............................................................ 7
SUPPLEMENTARY TABLE 3: BENCHMARKING OF ENSEMBLE CLASSIFIERS FOR RAMAN SPECTRA. 11
SUPPLEMENTARY FIG. 5: IMMUNE CELL TYPE SPECTRAL ANALYSIS....................................................... 12
SUPPLEMENTARY TABLE 4: SERS BAND ASSIGNMENTS FOR IMMUNE CELL SPECTRA........................13
SUPPLEMENTARY FIG. 6: PRE-SORTED CD19-CAR SPECTRAL ANALYSIS ACROSS DONORS................ 15
SUPPLEMENTARY FIG. 7: TRANSCRIPTOMIC SIGNATURES OF CAR+ VERSUS CAR- CELLS IN CD19 CAR T INFUSION PRODUCTS.........................................................................................................................16
SUPPLEMENTARY FIG. 8: NON-SERS CD19 CAR VS MOCK RAMAN DATA..................................................17
SUPPLEMENTARY FIG. 9: LOSS OF ANTI-IDIOTYPE LABEL AFTER POST-SORT INCUBATION...............18
SUPPLEMENTARY FIG. 10: POPULATION-LEVEL CO-CULTURE RAMAN SIGNATURES............................19
SUPPLEMENTARY FIG. 11: PSEUDOTIME TRAJECTORY ANALYSIS AND CORRELATION WITH REAL TIME..............................................................................................................................................................20
SUPPLEMENTARY FIG. 12: FLOW CYTOMETRY CHARACTERIZATION OF ANTIGEN-SPECIFIC ACTIVATION.................................................................................................................................................. 21
SUPPLEMENTARY FIG. 13: GD2-CAR AND MOCK SERS SPECTRAL CLASSIFICATION ACROSS DONORS.......................................................................................................................................................... 23
SUPPLEMENTARY FIG. 14: REPRESENTATIVE MITOCHONDRIAL MEASUREMENTS IN FMC-CAR, GD2-CAR, AND MOCK T CELLS................................................................................................................. 24
REFERENCES.....................................................................................................................................................25

**Supplementary Table 1:** Transduction efficiencies for all donors

| Donor ID | CAR construct | Transduction efficiency (%) |
|---|---|---|
| DN71 | CD19-CAR | N/A |
| DN74 | CD19-CAR | 96.1 |
| DN76 | CD19-CAR | 96.0 |
| DN81 | CD19-CAR | 76.0 |
| DN2058 | CD19-CAR | 71.7 |
| DN4400 | CD19-CAR | 78.3 |
| | GD2-CAR | 58.6 |
| DN4402 | CD19-CAR | 54.9 |
| | GD2-CAR | 41.2 |
| DN4408 | CD19-CAR | 55.8 |
| | GD2-CAR | 34.8 |
| DN4411 | CD19-CAR | 58.1 |
| | GD2-CAR | 41.4 |
| DN4414 | CD19-CAR | 59.1 |
| | GD2-CAR | 39.0 |
| DN7518 | CD19-CAR | 74.8 |

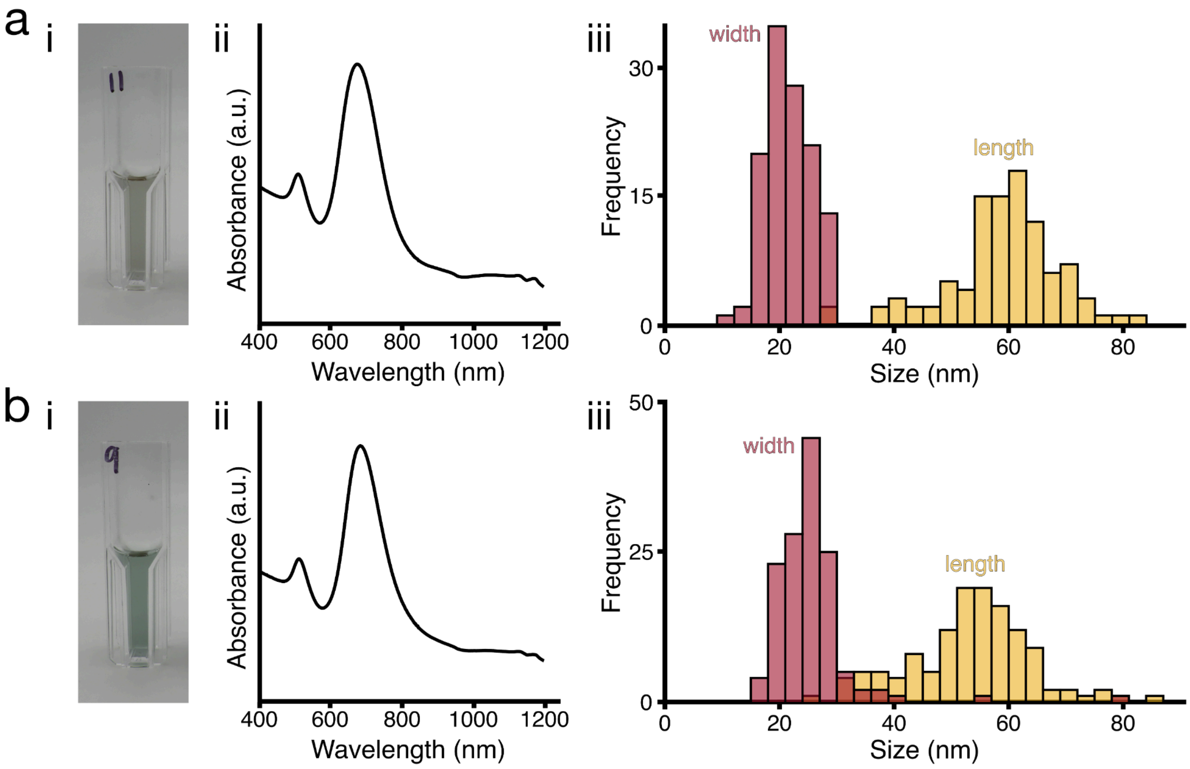


**Supplementary Fig. 1:** Gold nanorod characterization

Images of diluted nanorod suspensions (i), extinction spectra (ii), and size distributions (iii) for gold nanorods used for **(a)** donors DN71, 74, 76 (Fig. 3) and **(b)** all other donors. Both samples display a weak transverse plasmon resonance at ~520 nm and a strong longitudinal resonance at ~680 nm. Nanorods in (a) have an aspect ratio of 2.73 ± 0.66, and those in (b) have an aspect ratio of 2.13 ± 0.78.

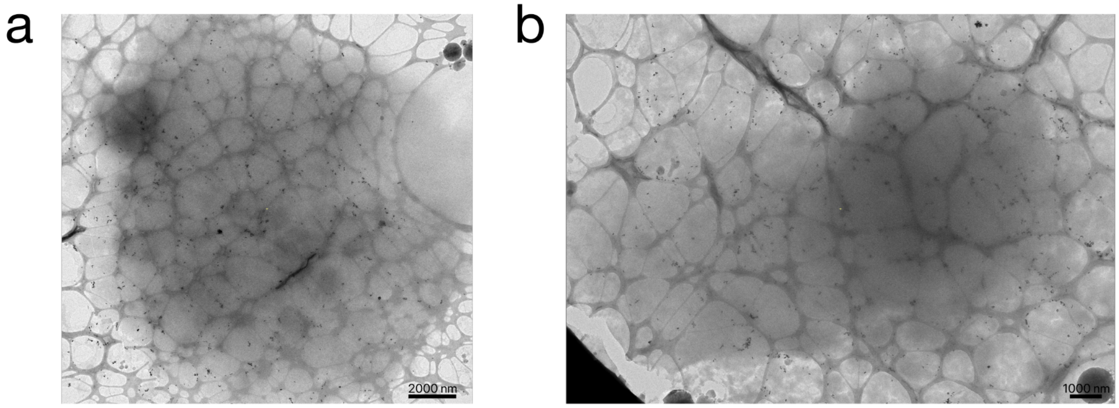


**Supplementary Fig. 2:** Cryo-electron micrographs of T cells with nanorods

Cryo-transmission electron micrographs of **(a)** a single frozen T cell and **(b)** two frozen T cells mixed with gold nanorods on lacey carbon grids. Smaller high-contrast features correspond to nanorods present on the cell surface and the grid. Supplementary Videos 1 and 3 show aligned cryo-tomography tilt series of the cells in (a) and (b), respectively, and Supplementary Video 2 includes a reconstructed tomogram of the cell in (a).

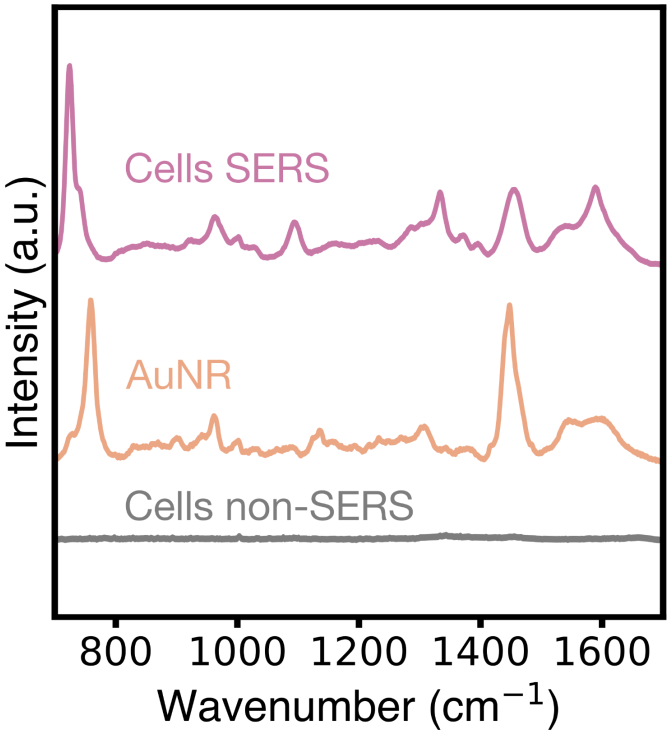


**Supplementary Fig. 3:** Cellular SERS vs. nanorod and non-SERS controls

Unnormalized Raman spectra of gold nanorods (AuNR, orange), cells without nanorods (cells non-SERS, grey), and cells with nanorods (cells SERS, pink), all taken with the same acquisition parameters. "Cells SERS" spectra exhibit significantly higher signal intensity than "cells non-SERS" spectra. Peaks in the AuNR spectrum likely arise from cetyltrimethylammonium bromide (CTAB) surfactant. The sharp bands in the AuNR spectrum, particularly the prominent 770 and 1445 cm$^{-1}$ features, are substantially reduced in the cellular SERS spectrum, consistent with minimal ligand contribution relative to cellular biomolecular signals.

**Supplementary Table 2:** Spectral count for each sample

| Class | Donor ID | Spectra count |
|---|---|---|
| **Figure 2: Blood Cell Type** | | |
| T cells | DN7518 | 1403 |
| | DN2058 | 900 |
| Primary B cells | — | 1911 |
| JeKo-1 B cells | — | 4965 |
| Red blood cells | — | 2318 |
| **Figure 3: CD19-CAR vs Mock (unsorted)** | | |
| CD19-CAR | DN71 | 1470 |
| | DN74 | 3555 |
| | DN76 | 4898 |
| | DN81 | 1002 |
| | DN4400 | 1093 |
| | DN4402 | 1096 |
| | DN4408 | 1078 |
| | DN4411 | 1121 |
| | DN4414 | 1121 |
| Mock | DN71 | 1352 |
| | DN74 | 2772 |
| | DN76 | 5840 |
| | DN81 | 1296 |
| | DN4400 | 1110 |
| | DN4402 | 1082 |

| | | |
|---|---|---|
| | DN4408 | 1087 |
| | DN4411 | 1109 |
| | DN4414 | 1091 |
| **Figure 3: CD19-CAR vs Mock (sorted)** | | |
| CD19-CAR | DN4400 | 1128 |
| | DN4408 | 1114 |
| | DN4411 | 1109 |
| | DN4414 | 1521 |
| Mock | DN4400 | 1211 |
| | DN4408 | 1122 |
| | DN4411 | 1114 |
| | DN4414 | 1553 |
| **Figure 4: Co-cultured CD19-CAR vs Mock** | | |
| CD19-CAR T & JeKo-1 B cells | DN7518 | 2321 |
| | DN2058 | 1442 |
| Mock T & JeKo-1 B cells | DN7518 | 2336 |
| | DN2058 | 1479 |
| **Figure 4: GD2-CAR vs Mock** | | |
| GD2-CAR | DN4400 | 1094 |
| | DN4402 | 1051 |
| | DN4408 | 1065 |
| | DN4411 | 1054 |
| | DN4414 | 1119 |
| | DN4400 | 1110 |

| | | |
|---|---|---|
| Mock | DN4402 | 1082 |
| | DN4408 | 1087 |
| | DN4411 | 1109 |
| | DN4414 | 1091 |
| **Supplementary Figure 7: non-SERS CD19-CAR vs Mock** | | |
| GD2-CAR | DN71 | 804 |
| | DN74 | 1121 |
| | DN76 | 1838 |
| Mock | DN71 | 1044 |
| | DN74 | 1246 |
| | DN76 | 1755 |

**Note**: Spectral counts are of analyzed spectra post-filtering for laser-induced damage or detector saturation (Supplementary Fig. 4).

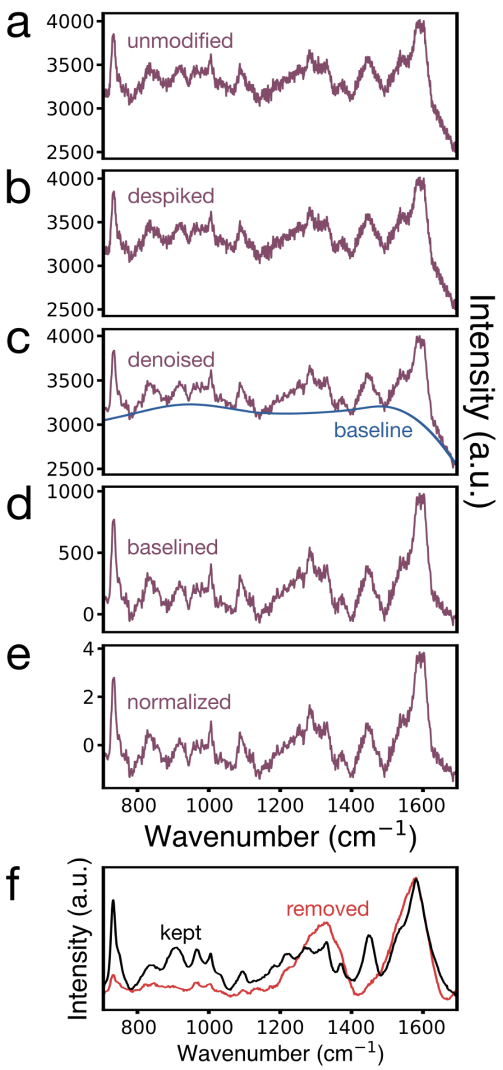


**Supplementary Fig. 4:** Raman spectral preprocessing workflow

A representative SERS spectrum of an immune cell shown **(a)** as acquired, **(b)** after cosmic ray removal,[1] **(c)** following wavelet-based denoising (baseline shown in blue),[2] **(d)** after adaptive iteratively reweighted penalized least squares baseline correction,[3] and **(e)** after normalization. **(f)** To exclude spectra impacted by laser-induced damage or detector saturation, spectra with average unnormalized intensities greater than 3 standard deviations above the mean for that experiment are removed. Shown are representative normalized average spectra retained (black) and removed (red) from one experiment.

**Supplementary Table 3:** Benchmarking of ensemble classifiers for Raman spectra

| **Model** | **F1** | **Accuracy** | **Train time (s)** | **Inference time (µs/sample)** |
|---|---|---|---|---|
| Random Forest | 0.71 ± 0.02 | 0.72 ± 0.02 | 8.1 ± 3.4 | 27.2 ± 9.8 |
| Support Vector Machine | 0.67 ± 0.00 | 0.50 ± 0.00 | 17.2 ± 2.8 | 2148.5 ± 106.0 |
| Explainable Boosting Machine | 0.75 ± 0.00 | 0.76 ± 0.00 | 291.5 ± 4.6 | 97.1 ± 3.1 |
| CatBoost | 0.74 ± 0.00 | 0.75 ± 0.00 | 53.0 ± 29.2 | 868.4 ± 326.0 |
| XGBoost | 0.73 ± 0.00 | 0.74 ± 0.00 | 27.5 ± 0.9 | 15.2 ± 2.8 |
| LightGBM | 0.74 ± 0.00 | 0.74 ± 0.00 | 9.5 ± 0.5 | 12.2 ± 1.0 |

**Note**: For a controlled comparison across models, we evaluated performance on a single-donor CD19-CAR vs Mock preprocessed Raman dataset (~1000 spectra/class) with added first- and second-derivative dimensions. All models were trained on the same stratified 80/20 train/test split, hyperparameters were tuned using an equal-budget randomized search (20 trials/model), and performance (accuracy and F1-score) was evaluated on the held-out test set.

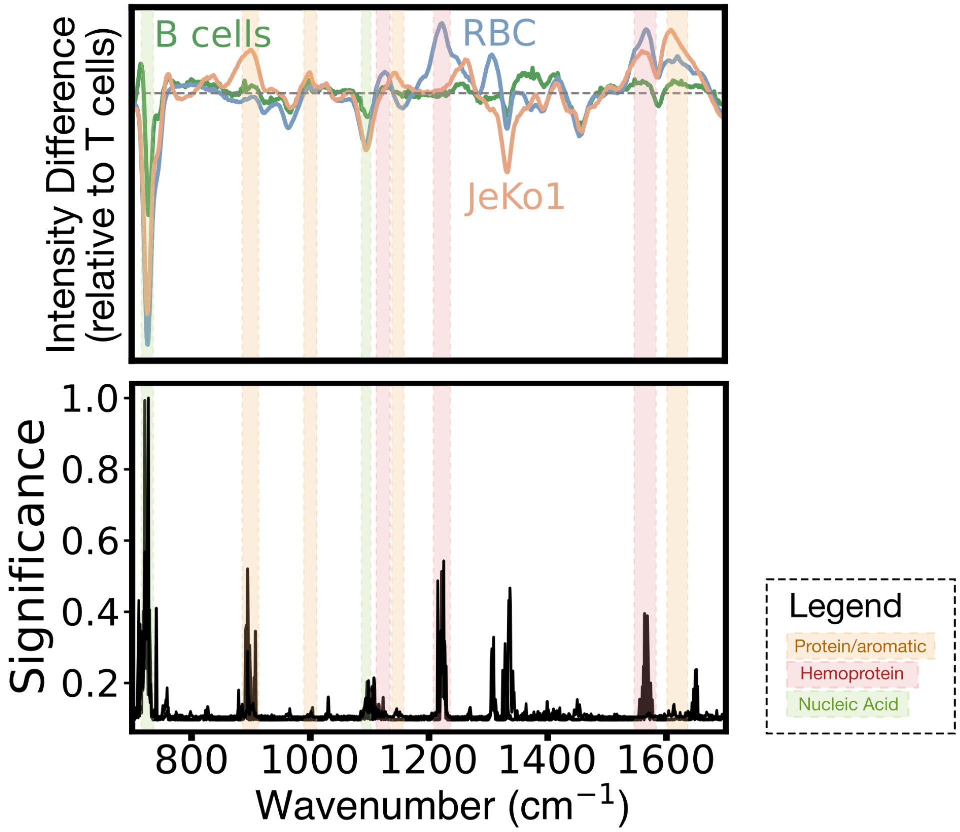


**Supplementary Fig. 5:** Immune cell type spectral analysis

Mean spectral difference of primary human B cells, JeKo-1 B cells, and red blood cells (RBC) relative to T cells (top). The grey dashed line marks zero difference. Feature importance plot showing the contribution of each spectral dimension (wavenumber) to classification among T cells, primary B cells, JeKo-1 B cells, and RBCs using a trained ML model (bottom). Colored spectral bands highlight protein/aromatic (gold), hemoprotein (red), and nucleic acid (green) regions.

**Supplementary Table 4:** SERS band assignments for immune cell spectra

| Wavenumber ($cm^{-1}$) | Assignment |
|---|---|
| 717-721 | Phosphocholine; symmetric C-N stretch[4] |
| 724 | Hypoxanthine; porphyrin ring mode[5] |
| 725-735 | Adenine; ring breathing mode[6–8] |
| 749-755 | Cytochrome c; heme breathing mode[9] |
| 870-880 | Phosphocholine; symmetric C-N stretch[4] |
| 880-900 | Tryptophan; indole ring mode[10,11] |
| 960-964 | Guanine[12] |
| 1,002-1,004 | Phenylalanine; ring breathing (C-C skeletal)[10,11] |
| 1,020-1,031 | Phenylalanine; C-N stretch[7] |
| 1,090-1,100 | Nucleic acid backbone; $PO_2^-$ symmetric stretch[6–9] |
| 1,127-1,129 | Hemoprotein; porphyrin ring mode[5] |
| 1,130-1,131 | Cytochrome c[9] |
| 1,140-1,160 | Protein; C-C and C-N stretch[6] |
| 1,175-1,177 | Aromatic amino acids (Tyr/Phe/Trp); C-H bending / scissoring[7,10] |
| 1,220-1,300 | Protein backbone; amide III region[10,11] |
| 1,225 | Deoxygenated hemoglobin[13] |
| 1,255-1,266 | α-helical protein; amide III region[10] |
| 1,310-1,311 | Cytochrome c[9] |
| 1,323-1,330 | Lipids; C-C, C-H stretches[9,11] |
| 1,330-1,346 | Nucleic acids (G/A/pyrimidines); ring C-N and C=N stretches[9,10,12] |
| 1,450-1,458 | Nucleic acids[12] |
| 1,455-1,465 | Lipids; $CH_2/CH_3$ deformation[6] |

| | |
|---|---|
| 1,480-1574 | Protein; amide II region[10,11] |
| 1,552-1,560 | Tryptophan; indole ring stretch[6,11] |
| 1,565-1,570 | Hemoprotein; porphyrin ring mode[5] |
| 1,584-1,590 | Cytochrome c[9] |
| 1,587-1,615 | Aromatic amino acids (Tyr/Phe/Trp); C=C/C=O stretches[7,10,11] |
| 1,620-1,625 | Hemoprotein; porphyrin ring $C_a$=$C_b$ C=C stretch[5] |
| 1,620-1,700 | Protein backbone; amide I region[10] |

**Note**: Raman peaks will vary based on cell type and culture conditions, SERS substrate (or lack thereof), and acquisition parameters such as excitation wavelength. Peak assignments should therefore be interpreted as approximate.

**Abbreviations:** Tyr: tyrosine; Phe: phenylalanine; Trp: tryptophan; G: guanine; A: adenine

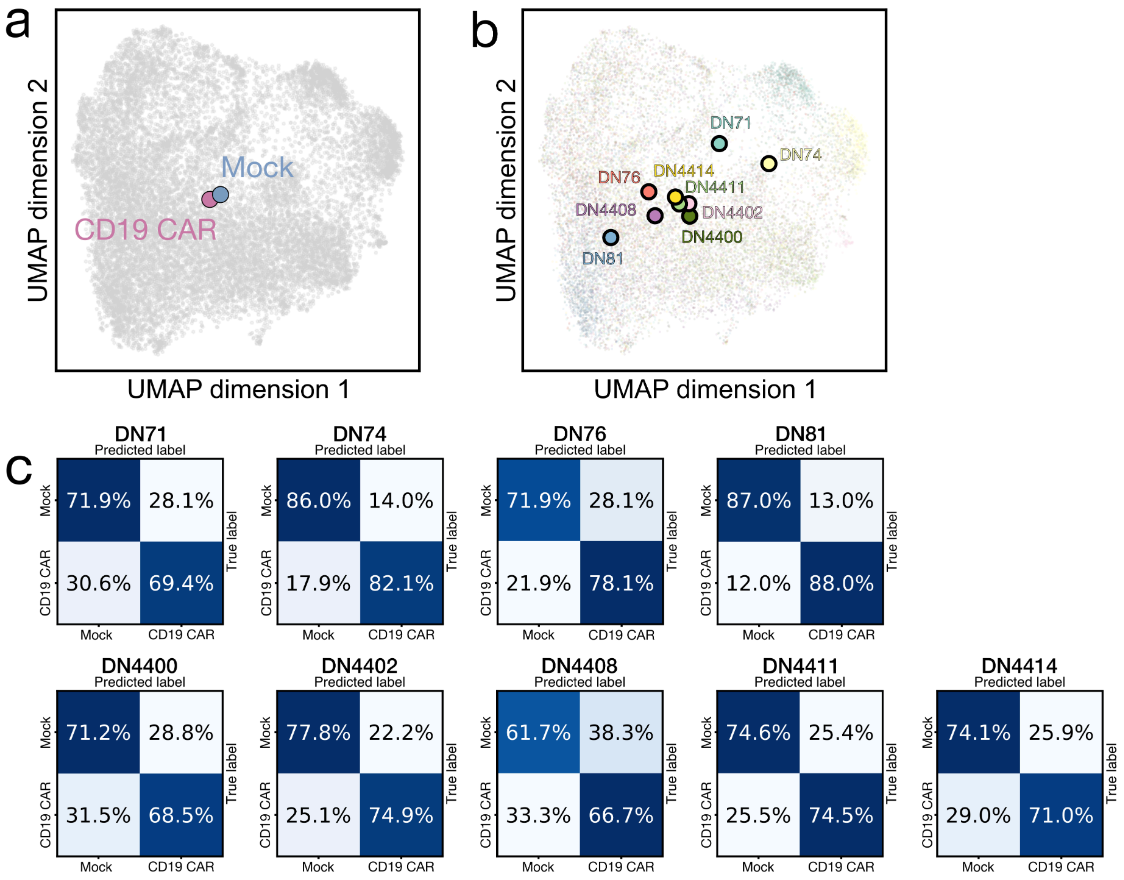


**Supplementary Fig. 6:** Pre-sorted CD19-CAR spectral analysis across donors

**(a-b)** Two-dimensional Uniform Manifold Approximation and Projections (UMAPs) of CD19-CAR and Mock Raman spectra from nine donors, generated after Principal Component Analysis (PCA) reduction and balanced by class and donor. **(a)** Class centroids and **(b)** donor centroids are overlaid, illustrating that inter-donor variability exceeds between-class separations. This strong donor prior motivated a per-donor model training for our dataset.[14] **(c)** Normalized confusion matrices for each donor showing classification accuracies for CD19-CAR and Mock spectra using a LightGBM classifier with 10-fold stratified cross-validation.

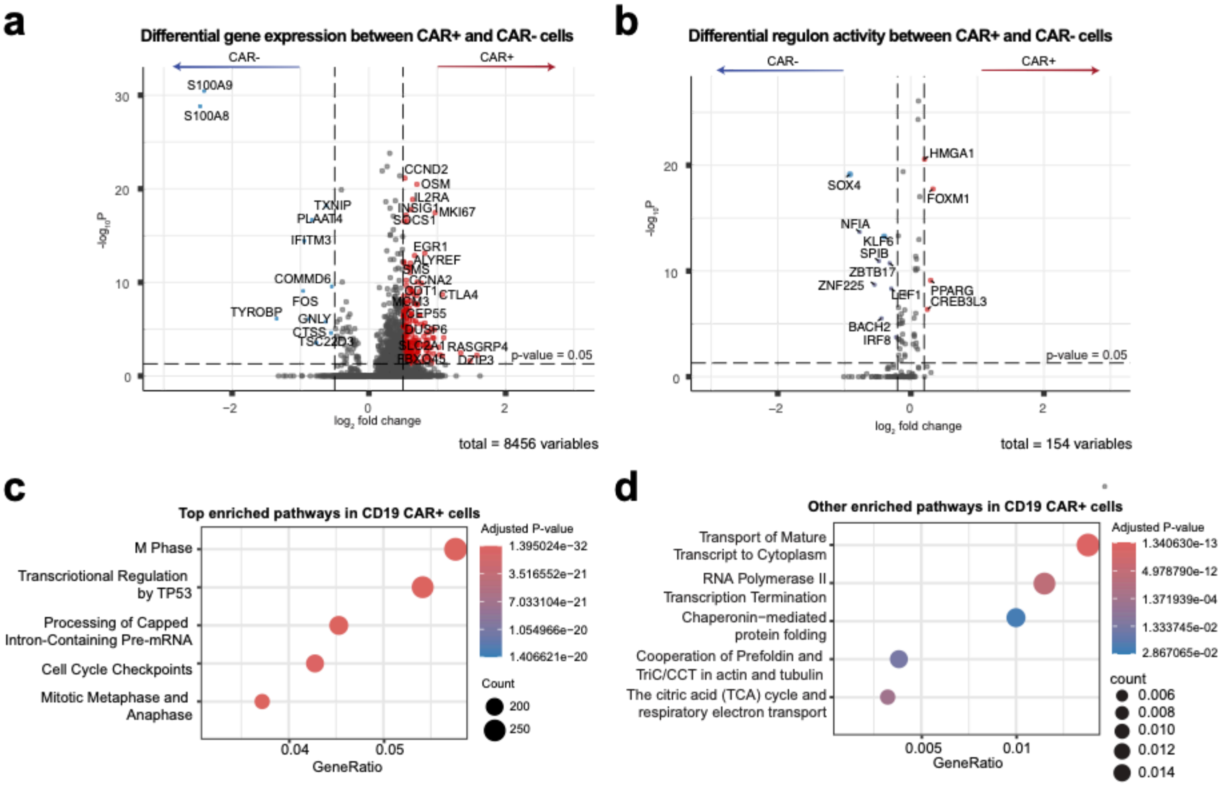


**Supplementary Fig. 7:** Transcriptomic signatures of CAR+ versus CAR- cells in CD19 CAR T infusion products

**(a)** Differential gene expression (DEG) volcano plot comparing CAR+ and CAR- infusion products. DEGs were selected based on log2 fold change |log2FC| > 0.3 and p-value < 0.05. **(b)** Differential regulon activity volcano plot comparing CAR+ and CAR- infusion products. **(c)** Dot plot showing top enriched pathways in CAR+ cells. **(d)** Additional enriched pathways in CAR+ groups, related to protein folding and aromatic expression.

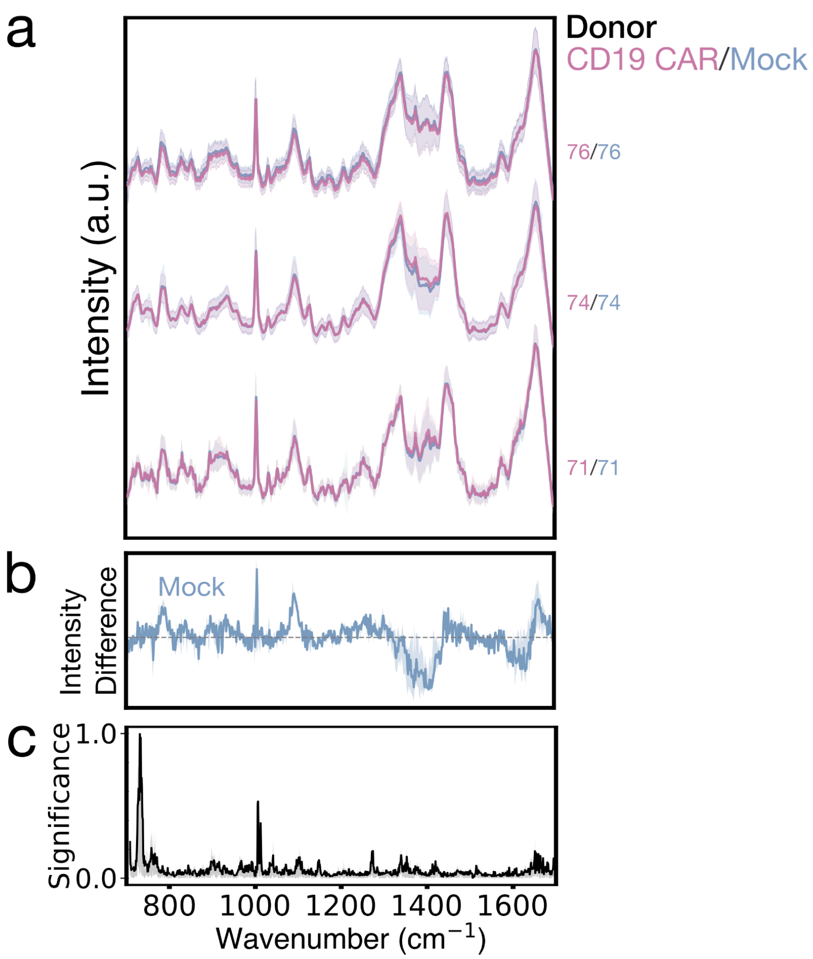


**Supplementary Fig. 8:** Non-SERS CD19 CAR vs Mock Raman data

**(a)** Waterfall plot of mean normalized spectra of live single CD19-CAR (pink) and Mock (blue) T cells with ±1 SD (shaded), separated by donor (identifier labeled on the right). **(b)** Median donor-normalized spectral difference of Mock cells relative to CAR with 25-75% quantile range. The grey dashed line marks zero difference. **(c)** Mean feature importance plot depicting the contribution of each spectral dimension (wavenumber) to classification using a trained LightGBM model.

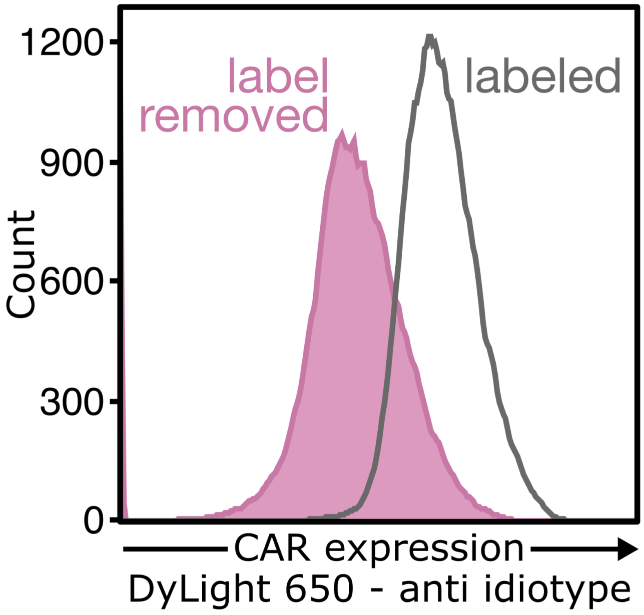


**Supplementary Fig. 9:** Loss of anti-idiotype label after post-sort incubation

Flow cytometry of purified CD19-CAR T cells following overnight incubation and washing. Cells analyzed without re-staining ("label removed", pink) show a substantial reduction in fluorescent signal compared to re-labeled cells with the same antibody ("labeled", grey). The decline in fluorescence post-incubation is consistent with loss of the initially bound antibody, potentially due to dissociation or internalization and degradation of the receptor-ligand complex. Re-labeling serves as a positive control confirming that CAR expression was maintained and the loss of signal in the "label removed" condition was due to anti-idiotype removal.

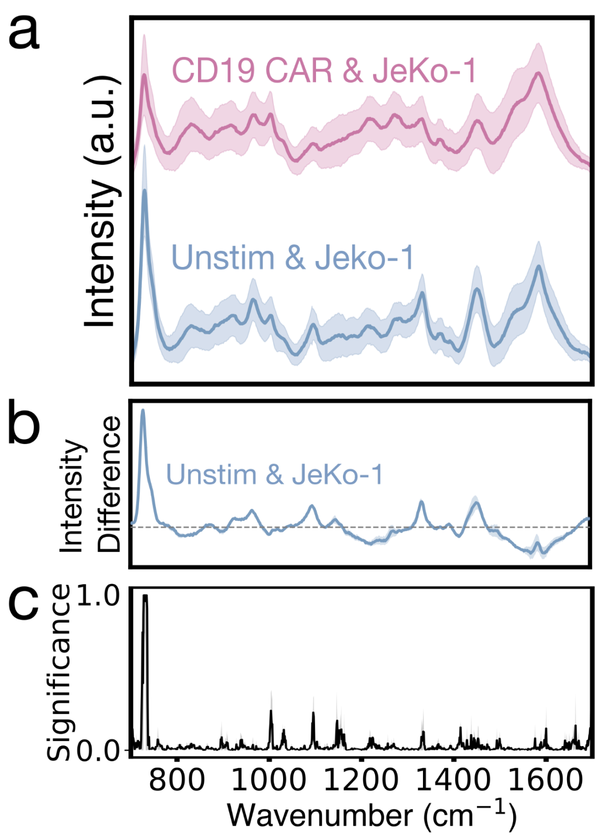


**Supplementary Fig. 10:** Population-level co-culture Raman signatures

**(a)** Mean normalized SERS spectra of live CD19-CAR (pink) and unstimulated (Unstim; blue) T cells co-cultured with JeKo-1 B cells, aggregated over the full 1.5-hour recording period (two donors), with ±1 s.d. (shaded). **(b)** Median donor-normalized spectral difference of Unstim cells relative to CAR T cells, with 25-75% quantile range. The grey dashed line marks zero difference. **(c)** Mean feature importance plot depicting the contribution of each spectral dimension (wavenumber) to classification using a trained LightGBM model.

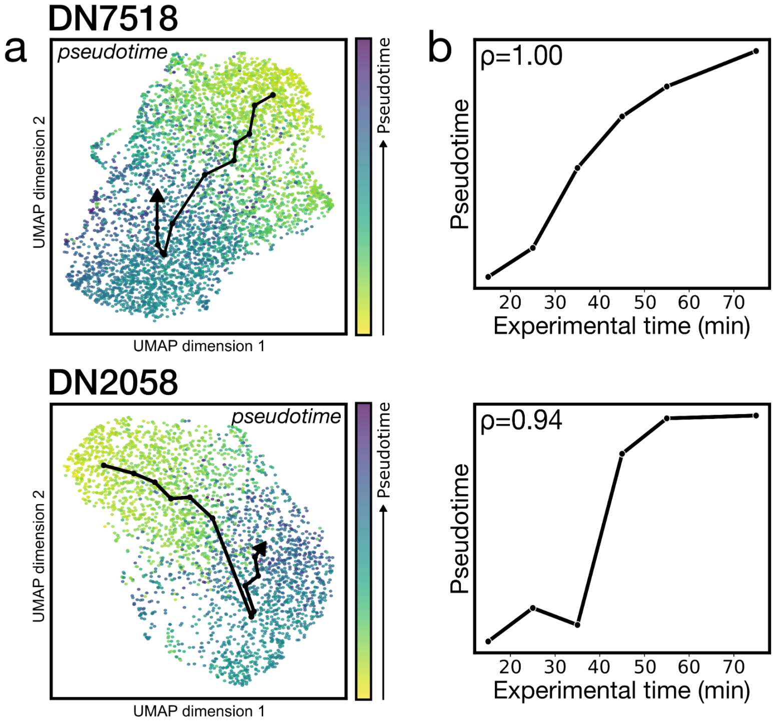


**Supplementary Fig. 11:** Pseudotime trajectory analysis and correlation with real time

**(a)** Pseudotime trajectories overlaid on UMAPs for two donors by pseudotime color scale (yellow to purple). The arrow indicates the trajectory direction backbone, connecting the centroids of pseudotime bins. **(b)** Comparison of experimental time and pseudotime across binned data revealed a strong correlation (Spearman's rank correlation of 1.00 for DN7518 and 0.94 for DN2058). Fluctuations from perfect monotonicity may be due to the activation heterogeneity across cells.

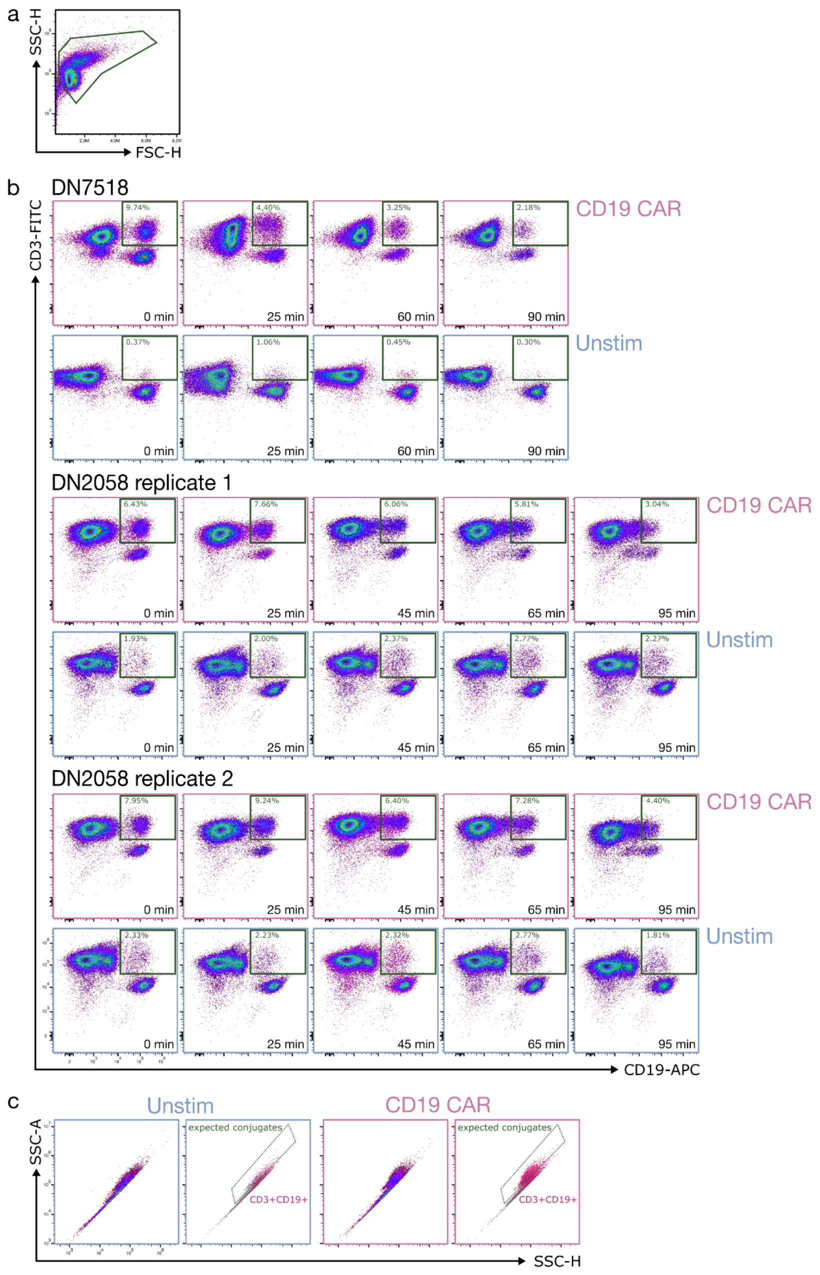


**Supplementary Fig. 12:** Flow cytometry characterization of antigen-specific activation

Flow cytometry density plots for CD19-CAR (pink) and unstimulated (Unstim; blue) T cells co-cultured with JeKo-1 B cells for approximately 90 minutes across two donors. **(a)** Density

flow plot showing the debris-removal gate (FSC-H vs SSC-H). No singlet/doublet gate was applied to preserve T-B cell conjugates in the CD3+CD19+ events. **(b)** CD3 vs CD19 density plots for all donors, replicates, and conditions across all measured time points, showing the strong presence of the CD3+CD19+ population (green gate) in the CD19-CAR condition, which is absent in the Unstim controls. **(c)** SSC-H vs SSC-A distributions for all events after debris removal in Unstim and CD19-CAR co-cultures at a representative timepoint (25 min) (left). CD3+CD19+ events overlaid (pink) on the full event distribution (grey). In the CD19-CAR condition, these events localize to a high-side-scatter region ("expected conjugates"), consistent with interacting cells, whereas Unstim samples show few such events.

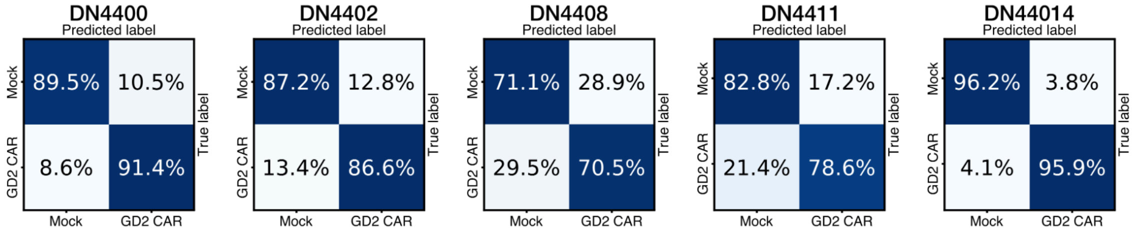


**Supplementary Fig. 13:** GD2-CAR and Mock SERS spectral classification across donors

Normalized confusion matrices for five donors showing classification accuracies for GD2-CAR and Mock spectra using a LightGBM classifier with 10-fold stratified cross-validation.

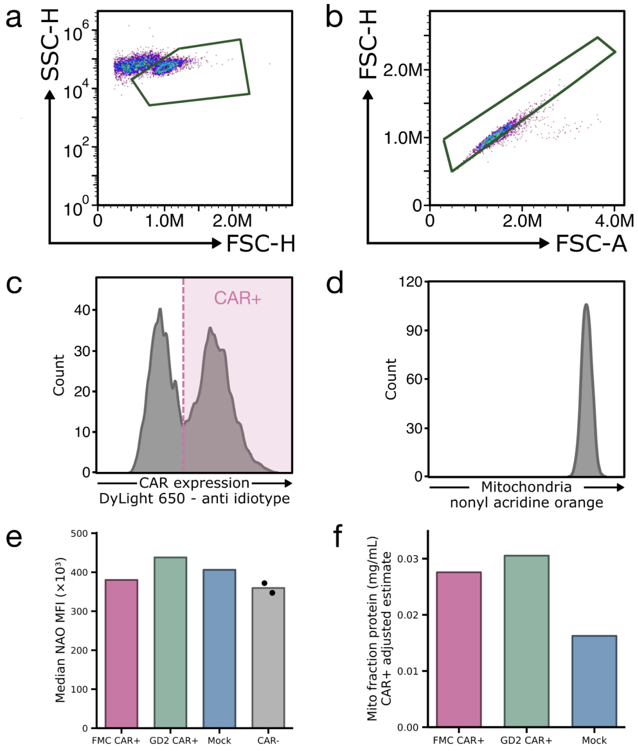


**Supplementary Fig. 14:** Representative mitochondrial measurements in FMC-CAR, GD2-CAR, and Mock T cells

Flow cytometry gating strategy for mitochondrial mass quantification. Flow cytometry plots show gating for **(a)** lymphocytes, **(b)** singlets, and **(c)** CAR-expressing cells with CAR+ gate indicated (anti-CAR staining; DyLight 650). **(d)** Mitochondrial mass was assessed using nonyl acridine orange (NAO). **(e)** Median NAO fluorescence intensity (MFI) is plotted for CAR-positive FMC-CAR and GD2-CAR T cell populations, the full Mock T cell population, and CAR-negative cells (negative gate) from the CAR-transduced samples (representative donor). **(f)** Representative mitochondrial extraction followed by Bradford assay quantification of mitochondrial protein concentration. For CAR-transduced samples, concentrations were adjusted by the CAR+ transduction efficiency measured using flow cytometry to estimate the protein concentration per CAR+ cell. Mock was unadjusted.

# References


1. Whitaker, D. A. & Hayes, K. A simple algorithm for despiking Raman spectra. *Chemometr. Intell. Lab. Syst.* **179**, 82–84 (2018).
2. Chang, S. G., Yu, B. & Vetterli, M. Adaptive wavelet thresholding for image denoising and compression. *IEEE Trans. Image Process.* **9**, 1532–1546 (2000).
3. Zhang, Z.-M., Chen, S. & Liang, Y.-Z. Baseline correction using adaptive iteratively reweighted penalized least squares. *Analyst* **135**, 1138–1146 (2010).
4. Akutsu, H. Structure and dynamics of phospholipids in membranes elucidated by combined use of NMR and vibrational spectroscopies. *Biochim. Biophys. Acta Biomembr.* **1862**, 183352 (2020).
5. Premasiri, W. R., Lee, J. C. & Ziegler, L. D. Surface-enhanced Raman scattering of whole human blood, blood plasma, and red blood cells: cellular processes and bioanalytical sensing. *J. Phys. Chem. B* **116**, 9376–9386 (2012).
6. Chaudhary, N., Nguyen, T. N. Q., Cullen, D., Meade, A. D. & Wynne, C. Discrimination of immune cell activation using Raman micro-spectroscopy in an in-vitro & ex-vivo model. *Spectrochim. Acta A Mol. Biomol. Spectrosc.* **248**, 119118 (2021).
7. Chan, J. W. *et al.* Micro-Raman spectroscopy detects individual neoplastic and normal hematopoietic cells. *Biophys. J.* **90**, 648–656 (2006).
8. Borek-Dorosz, A. *et al.* Raman-based spectrophenotyping of the most important cells of the immune system. *J. Adv. Res.* **41**, 191–203 (2022).
9. Borek-Dorosz, A. *et al.* Alterations in lipid metabolism accompanied by changes in protein and carotenoid content as spectroscopic markers of human T cell activation. *Biochim. Biophys. Acta Mol. Cell Biol. Lipids* **1869**, 159496 (2024).

10. Parker, F. S. Biochemical applications of infrared and Raman spectroscopy. *Appl. Spectrosc.* **29**, 129–147 (1975).
11. Stepanenko, T. *et al.* Surface-enhanced Raman scattering (SERS) and tip-enhanced Raman scattering (TERS) in label-free characterization of erythrocyte membranes and extracellular vesicles at the nano-scale and molecular level. *Analyst* **149**, 778–788 (2024).
12. Safar, W., Azziz, A., Edely, M. & Lamy de la Chapelle, M. Conventional Raman, SERS and TERS studies of DNA compounds. *Chemosensors (Basel)* **11**, 399 (2023).
13. Atkins, C. G., Buckley, K., Blades, M. W. & Turner, R. F. B. Raman spectroscopy of blood and blood components. *Appl. Spectrosc.* **71**, 767–793 (2017).
14. Ciobanu, C. *et al.* Exploring the use of Raman spectroscopy and covariate-adjusted multivariate analysis for the detection of irradiated blood. *Radiat. Res.* **199**, 396–405 (2023).
